# Retinal proteome changes mirror brain pathology and reveal synaptic and cytoskeletal dysfunction in Alzheimer’s disease

**DOI:** 10.1007/s00401-026-03054-x

**Published:** 2026-07-29

**Authors:** Jessica Santiago, Dovilė Pocevičiūtė, Teo Sällberg, Patrik Önnerfjord, The Netherlands Brain Bank, Jacob W. Vogel, Malin Wennström

**Affiliations:** 1 Cognitive Disorder Research Unit, Department of Clinical Sciences Malmö, Lund University, Inga Marie Nilssons Gata 53, 214 28 Malmö, Sweden; 2 Center for Translational Proteomics, Department of Clinical Sciences Lund, Lund University, Lund, Sweden; 3 Netherlands Institute for Neuroscience, Amsterdam, The Netherlands; 4 Department of Clinical Sciences Malmö, SciLifeLab, Lund University, Lund, Sweden

**Keywords:** Retina, Alzheimer’s disease, Proteomics

## Abstract

Visual dysfunction is increasingly recognized as an important feature of Alzheimer’s disease, and substantial retinal changes have been documented across multiple studies. Yet the molecular changes underlying retinal neurodegeneration and which retinal protein signatures best track cerebral pathology remain incompletely defined. Here, we performed comprehensive mass spectrometry-based proteomics on paired retinal and hippocampal tissue from the same postmortem donors (8 AD, 8 non-demented controls) to identify disease-associated molecular signatures and assess their overlap between these tissues. Using a sequential dual-extraction protocol, we identified 372 differentially abundant retinal proteins in AD, including established APP-processing regulators (SORL1, BACE1) and synaptic proteins. Retinal proteomes clearly separated AD from controls in principal component analysis, indicating robust AD-related molecular differences in the retina. Notably, 87% of proteins were detected in both retina and hippocampus, with 64 differentially abundant proteins shared between tissues, some of which showed strong cross-tissue correlation. Several retinal proteins also correlated with neuropathological disease stage. Functional enrichment analysis revealed convergent alterations in synaptic organization, cytoskeletal dynamics, mitochondrial function, cell adhesion, and APP metabolism in both tissues. Cell-type mapping using single-cell retinal reference data indicated that most proteomic changes were broadly distributed across cell types, though some proteins showed enrichment in specific populations, such as SORL1 in microglia and EYS in photoreceptors. The molecular changes identified here offer a potential basis for the retinal alterations previously documented through in vivo imaging and histological studies. Their similarities with brain pathology further support the retina as a promising window for assessing cerebral disease.

**Supplementary Information:**

The online version contains supplementary material available at 10.1007/s00401-026-03054-x.

## Introduction

Alzheimer’s disease (AD) is the leading cause of dementia worldwide, affecting about one in nine people aged 65, with prevalence rising as populations age [1]. Characterized by the accumulation of amyloid-β (Aβ) and hyperphosphorylated tau [11, 12, 17, 63], AD is now recognized as a multifactorial neurodegenerative disorder. Many pathological processes, including inflammation, blood–brain barrier dysfunction, and synaptic loss, start up to 2 decades before cognitive symptoms begin [5, 8, 30, 56, 59, 83, 84]. Proteomic analyses of brain and CSF reveal that AD involves a coordinated remodeling of the proteome, where metabolic and mitochondrial disturbances are linked with alterations in neuronal structure and glial activity [32, 53, 72, 81].

Although cognitive decline is the defining clinical feature of AD, the pathology is not restricted to the brain, and retinal involvement is increasingly recognized as a prominent aspect of the disease [1, 19]. Histological examination and retinal imaging have shown that AD-associated changes are accompanied by marked structural alterations in the retina: key findings include degeneration of retinal ganglion cells, thinning of the retinal nerve fiber layer, and vascular abnormalities [9, 15, 16, 19, 35, 46, 66]. These structural alterations likely contribute to the visual impairments observed in some AD patients [3, 79], which have been associated with reduced quality of life and accelerated cognitive decline [21, 54, 73].

At the molecular level, postmortem studies have reported accumulation of Aβ and hyperphosphorylated tau in AD retinas, alongside other amyloidogenic proteins [18, 22, 24, 38, 49, 60–62]. These deposits appear to coincide with a broad glial response, including reactive astrogliosis and activation of microglia and Müller cells [22, 49, 78]. Retinal vascular pathology has also been reported with marked signs of blood–retinal barrier disruption and capillary degeneration [64, 66]. In addition, previous studies have also implied changes in cytoskeletal integrity and mitochondrial function associated with Aβ accumulation [20]. A previous study investigating proteomic changes in the AD retina reported an increase in proteins associated with inflammation and neurodegeneration, along with a downregulation of proteins linked to mitochondrial function and photoreceptor integrity [34].

Despite this growing body of evidence, key questions remain about the molecular alterations underlying retinal neurodegeneration and how these changes relate to cerebral pathology. No study has directly compared retinal and brain proteomes from the same individuals or systematically mapped pathway-level alterations across both tissues in AD. Given the retina’s unique accessibility for non-invasive assessment of neurons, glia, and vasculature in vivo [7, 41], resolving these questions is essential for establishing its potential as a molecular window into the disease.

**Fig. 1:**
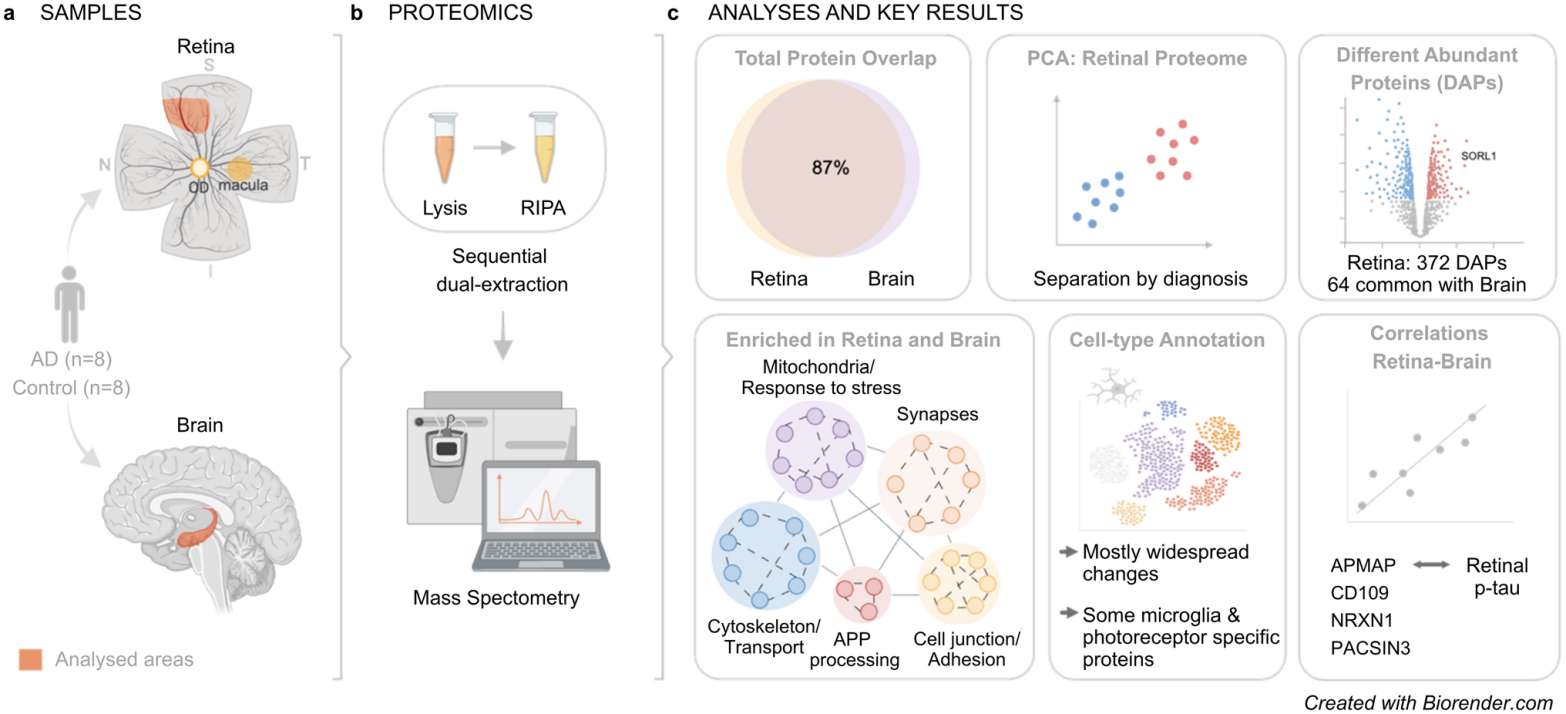
Schematic overview of the study design, proteomics workflow, and key findings. **a** Postmortem retina and hippocampus tissues from Alzheimer’s disease (AD; *n* = 8) patients and non-demented controls (NC; *n* = 8) were collected for analysis. **b** Proteins were sequentially extracted using a two-step approach, consisting of an initial Lysis buffer extraction followed by RIPA buffer extraction, and analyzed by mass spectrometry. **c** Bioinformatics analyses revealed a distinct AD-associated protein signature in the retina and substantial proteomic overlap between retina and brain. Pathway analysis identified dysregulation of common molecular processes, including synaptic signaling, cytoskeletal transport, and APP processing. Retinal proteome changes were widespread across cell types, with enrichment of some microglia and photoreceptor-associated proteins. Cross-tissue correlations identified shared protein alterations between retina and hippocampus and associations with retinal phosphorylated tau (p-tau)

To contribute toward answering these questions, we designed a mass spectrometry-based proteomics study analyzing postmortem retinas and hippocampi from the same individuals. We hypothesize that the retina, which shares neurons, glia, and an analogous vascular barrier with the brain [41], undergoes similar AD-associated molecular alterations. Specifically, we expect dysregulation of inflammatory, mitochondrial, and synaptic pathways. This paired design, combined with a sequential proteomic approach to capture proteins of varying solubilities and from diverse cellular compartments, provides a comprehensive view of retinal pathology and enables direct comparison of molecular changes in the retina and brain within each subject. Our study has two principal aims: (1) to identify protein expression and pathway alterations in AD versus non-demented control (NC) retinas and (2) to assess the similarities between retinal and brain molecular changes within the same individuals. An overview of the study design, analytical workflow, and principal findings is presented in Fig. 1.

## Materials and methods

### Tissue samples and donor information

Paired retinal and hippocampal postmortem tissue samples were obtained from The Netherlands Brain Bank (NBB), comprising neuropathologically confirmed AD (*n* = 8) and NC (*n* = 8). Hippocampal tissue was unavailable for one NC donor; consequently, all retinal analyses were performed on the full cohort (AD *n* = 8, NC *n* = 8), while brain analyses and cross-tissue comparisons were restricted to donors with paired tissue available (AD *n* = 8, NC *n* = 7). Individuals with a diagnosis of macular degeneration were excluded, and none of the participants had other significant ophthalmological conditions, including glaucoma or diabetic retinopathy. Detailed demographic and clinical characteristics of the study cohort are provided in Table 1 and Supplementary Table 1.

**Table 1:** Cases included in the study

	NC (*n* = 8)	AD (*n* = 8)
Age, years (mean ± SD)	79.0 ± 13.7	78.2 ± 13.0
Gender, M/F (% female)	4/4 (50%)	2/6 (75%)
Postmortem delay, minutes (mean ± SD)	362 ± 79	360 ± 56
APOE4 positive, *n* (%)	3 (38%)	5 (62.5%)
Neurofibrillary tangle score (median, range)	1.5 (0–3)	5.0 (4–6)
Amyloid-β score (median, range)	0 (0–3)	3 (2–3)

Data are shown as mean ± SD for age and postmortem delay, *n* (%) for gender and APOE4 status, and median (range) for NFT and Aβ scores

Neuropathological evaluation followed Braak and Braak (1991) [11]. Neurofibrillary changes were staged using the classical six Braak stages (I–VI). Amyloid-β (Aβ) plaques were assessed according to the cortical distribution as described in the original paper: O (0, no detectable amyloid), A (1, sparse plaques restricted to association cortices), B (2, numerous plaques in association areas with occasional involvement of primary cortices), and C (3, abundant plaques throughout association, belt, and core/primary cortical fields) [11]. Although this Aβ staging method is not commonly applied today, it was the classification approach used by the biobank at the time of autopsy.

Written informed consent for the use of tissue and clinical data in research was obtained from all donors or their legal representatives, in accordance with the Declaration of Helsinki and the European Code of Conduct for Brain Banking. The tissue collection protocol was approved by the Medical Ethical Committee of the VU Medical Center, Amsterdam, and all research procedures were reviewed and approved by the regional ethical review board in Lund.

### Processing of retinal and hippocampal samples for mass spectrometry

At autopsy, the lenses of the right eyes were removed, filled with O.C.T. mounting medium (Vector Laboratories), and subsequently frozen for storage at − 80 °C. Each eyeball was cut into eight clefts, leaving a 0.5 cm margin around the optic nerve intact. Retinal tissue was collected from the superior-nasal region for this study. The superior retina was selected based on converging evidence that it is among the most affected regions in AD: it shows pronounced thinning of the retinal nerve fiber layer relative to other quadrants [19], greater phosphorylated tau accumulation compared to medial retina in postmortem tissue[18], and has been identified as a hotspot for Aβ plaque deposition in whole-mount retinal analyses of AD patients[33]. The specific selection of the superior-nasal sub-region ensured consistent and reproducible dissection across donors.

Hippocampal tissue was snap-frozen at autopsy in 1-cm-thick sections and stored at − 80 °C. For analysis, the tissue was further sectioned into 0.5-cm-thick slices. A 3 mm biopsy punch (Kai Medical) was used to dissect samples from the Cornu Ammonis 1 (CA1) region adjacent to the subiculum. The CA1 was selected due to its vulnerability to AD pathology [11, 36].

Both retinal and hippocampal samples were transferred to 1.5 mL Pink Rino Tubes with screw caps, and 100 µL of Lysis buffer (50 mM Tris–HCl, pH 7.5, 50 mM NaCl, 1 mM EDTA, 5 mM NaH₂PO₄, 1 mM DTT, 0.1% phosphatase 1, 0.03% phosphatase 2, 0.05% protease inhibitors; Sigma-Aldrich) was added. Samples were homogenized in a Bullet Blender Storm Pro (BT24M, Next Advance, Inc., Troy, NY, USA) at speed 8 for 3 min, followed by centrifugation at 14,000×g for 10 min. Supernatants were collected into new Eppendorf tubes. The residual material was washed with 50 µL Lysis buffer, centrifuged again, and the supernatant pooled with the previous fraction. The remaining tissue in the Rino tubes underwent a second extraction using RIPA buffer (50 mM Tris–HCl pH 7.4, 150 mM NaCl, 1 mM EDTA, 1% Triton X-100, 0.1% sodium deoxycholate) and supernatants were collected in new Eppendorf tubes.

This two-step protocol was designed to capture proteins with different biochemical properties and solubilities. The Lysis buffer uses mild ionic conditions without detergents, extracting soluble cytoplasmic proteins and loosely membrane-associated proteins while mostly preserving cellular structures. The subsequent RIPA buffer employs both ionic and non-ionic detergents (Triton X-100 and sodium deoxycholate) under higher ionic strength, enabling the extraction of proteins from disrupted membranes, protein complexes, and cellular compartments resistant to mild Lysis. This sequential approach allows detection of proteins that might exist in different biochemical states (e.g., soluble vs. complex-bound) or subcellular localizations between disease and control tissues, providing complementary views of the proteome that would not be captured by a single extraction method.

For downstream processing, 50 µL of each sample was reduced with DTT (final 10 mM) at 56 °C for 30 min, followed by alkylation with iodoacetamide (final 20 mM) for 30 min at RT in the dark. Proteins were precipitated with ice-cold ethanol (final 90%) overnight at − 20 °C and pelleted by centrifugation at 14,000×g for 10 min. Pellets were air-dried and resuspended in 50 µL 100 mM ammonium bicarbonate, then disrupted using a BioRuptor (Diagenode Inc., Denville, USA) for 20 cycles of 15 s on/off. Samples were centrifuged at 14,000×g for 10 min, and the supernatant was transferred to new tubes. Protein concentration was determined using a NanoDrop (DeNovix, AH Diagnostics) at A₂₈₀ nm.

For digestion, 15 µg of protein per sample was incubated with trypsin (Promega, Madison, WI) at a 1:50 enzyme:protein ratio overnight at 37 °C. Digestion was stopped by adding 5 µL of 10% trifluoroacetic acid (TFA). Samples were dried in a SpeedVac and resuspended in 22 µL of 2% acetonitrile/0.1% TFA for mass spectrometry analysis.

### Liquid chromatography–tandem mass spectrometry (LC–MS/MS) analysis

Peptide separation and mass spectrometry: For analysis, 2 µL of each sample was injected into an Exploris 480 mass spectrometer (Thermo Fisher Scientific) coupled to a Vanquish Neo UHPLC system (Thermo Fisher Scientific). Peptides were first loaded onto an Acclaim PepMap 100 C18 precolumn (75 µm × 2 cm, Thermo Scientific) and then separated on an EASY-Spray C18 column (75 µm × 25 cm, 2 µm, 100 Å, ES902) at a flow rate of 300 nL/min and a column temperature of 45 °C. A 120 min nonlinear gradient was applied using Solvent A (0.1% FA in water) and Solvent B (0.1% FA in 80% ACN): 5–25% B over 100 min, 25–32% B over 12 min, and 32–45% B over 8 min.

Mass spectrometry acquisition: Data were acquired in data-dependent acquisition (DDA) mode in positive polarity. Full MS1 scans were acquired at a resolution of 120,000 (m/z 200), with a normalized AGC target of 300% and a maximum injection time of 45 ms over a mass range of 350–1400 m/z. Precursors were isolated using a 1.3 m/z window and fragmented by HCD with a normalized collision energy of 30. MS2 spectra were recorded in the Orbitrap at a resolution of 15,000, with a normalized AGC target of 100% and custom maximum injection time. An intensity threshold of 10^4^ and dynamic exclusion of 60 s were applied.

Raw spectra were processed using Proteome Discoverer 2.5 (Thermo Fisher Scientific) and searched against the UniProt Human canonical database (UP000005640) for protein identification and label-free quantification. Here, the retinal and hippocampal samples were analyzed in a single integrated workflow to enable direct cross-tissue comparisons within individual subjects. Precursor and fragment tolerances were set to 10 ppm and 0.02 Da, respectively. Trypsin was specified as the protease, with methionine oxidation, asparagine deamidation, phosphorylation (STY), oxidation (M), and protein N-terminal acetylation set as variable modifications, and cysteine carbamidomethylation as a fixed modification. Label-free quantification was performed using peptide peak intensities extracted from Proteome Discoverer.

### Preprocessing

Label-free protein intensities (obtained via Proteome Discoverer 2.5) were first filtered to retain proteins identified at ≤ 1% FDR, supported by at least two unique peptides, and detected in a minimum of 70% of samples. After log₂ transformation and median normalization per sample (row-wise), missing values were imputed using a left-shifted, normal distribution (shifted 2 SD below the protein’s mean log-intensity, with a width of 0.3 × SD, capped at the lowest observed value). This models undetected low-abundance signals and is standard for Missing-Not-At-Random (MNAR) imputation in label-free proteomics [26, 37].

### Immunofluorescence staining

Flatmounts from the far periphery of the nasal superior retina of the right eye from a subset of the (*n* = 6) AD and (*n* = 6) NC cases were mounted on Omnipore membranes (Merck) and cross-sectioned into 40 µm. The sections were then photobleached overnight with a 450 nm–460 nm blue LED light at 4 °C according to a previously described protocol [43] to omit autofluorescence. The membranes with the cross-sections were washed with phosphate-buffered saline (PBS) three times and incubated in blocking solution (BS) consisting of 5% goat serum and 0.25% triton in PBS for 1 h at room temperature (RT). Thereafter, the primary antibodies were added to the BS, and the samples were left overnight at 4 °C on a slow shaker. The following primary antibodies were used, each on separate sections: rabbit anti-SORL1 (Abcam, clone EPR23262-4,1:300), rabbit anti-APMAP (Sigma-Aldrich, Cat#HPA012863,1:300), or rabbit anti-PACSIN3 (Proteintech, Cat#10639-1-AP,1:300).

The day after the samples were rinsed six times in PBS + 0.25% triton and subsequently incubated with Goat anti-Rabbit 549 (Invitrogen) and DAPI for 2 h at RT. Finally, the membranes with the cross-sections were mounted on glass slides using Vectashield Set mounting medium (Vector Laboratories). A negative control for the retinal staining was included in the immunostaining procedure by omitting the primary antibody, thereby ruling out nonspecific binding of the secondary antibody or background staining.

Images of the immunostainings of the retina were captured using a confocal microscope (Zeiss LCM 800) with a 63 × objective and an Olympus AX70 light microscope with a 20 × objective. DAPI staining was used to guide the identification of retinal layers. For quantification, ROIs corresponding to anatomical regions of interest were defined in the DAPI channel and applied to the target channel in Fiji (ImageJ). All images were converted to 16-bit prior to analysis, and the background was subtracted. Signal intensity was then quantified as the thresholded area fraction, using a consistent threshold across all images. Three images per retina were acquired, and the values were averaged per subject before statistical analysis.

### Statistical analyses

Differential abundance between AD and NC samples was assessed separately in the retina and hippocampus using linear mixed-effects modeling (via statsmodels MixedLM). The fixed-effects structure was specified as:

$${\text{Protein }}\sim {\text{ C}}\left( {{\mathrm{Buffer}}} \right) \, *{\text{ C}}\left( {{\mathrm{Diagnosis}}} \right) \, + {\text{ C}}\left( {{\mathrm{Sex}}} \right) \, + {\text{ Age }} + \left( {1|{\mathrm{Subject}}} \right)$$

where *Buffer* denotes the extraction method (Lysis or RIPA), *Diagnosis* indicates AD versus NC, *Sex* and *Age* were included as covariates to control for demographic differences, and *Subject* identity was modeled as a random intercept to account for paired measures (both buffers from the same donor).

For both tissues, the primary focus was on the main effect of Diagnosis; the Buffer × Diagnosis interaction was evaluated as a secondary outcome to explore whether diagnostic effects differed between extraction conditions, reflecting potential differences in protein solubility or subcellular compartmentalization. The regression coefficient for diagnosis (β) reflects the magnitude and direction of protein abundance differences between AD and control samples, expressed on a log2 scale. *P* values were corrected for multiple testing using the Benjamini–Hochberg false discovery rate (FDR) procedure, and proteins with FDR < 0.05 were considered differentially abundant (DAPs). The intersection of DAPs between the retina and hippocampus was identified for downstream comparative analyses.

Partial Spearman correlation analyses (adjusted for age and sex) were conducted to examine disease-related associations. First, correlations were computed between each of the top 20 upregulated and downregulated retinal proteins (based on FDR-adjusted *P *values) and stages of Alzheimer’s pathology, neurofibrillary tangle (NFT), and amyloid β (Aβ) plaque burden. Second, proteins that were differentially abundant in both retina and brain and exhibited changes in the same direction and with an effect size of |β|≥ 1.0 were correlated between tissues to identify shared molecular alterations. For all correlation analyses, results were also evaluated based on FDR-adjusted values (Benjamini–Hochberg), and leave-one-out (LOO) analyses were performed to assess the robustness of findings.

Immunofluorescence signal intensities were log-transformed, and group differences between AD and controls were assessed using ordinary least squares regression on rank-transformed values, adjusting for age and sex. Results are reported as beta coefficients with 95% confidence intervals. Plots were generated in Python using seaborn and matplotlib.

### Enrichment and STRING analyses

Functional enrichment of differentially abundant proteins (DAPs) was performed using g:Profiler, examining Gene Ontology (BP, MF, CC), and Reactome pathways. Analyses were conducted using two complementary background sets: (i) the full proteome detected in the experiment, to account for proteins actually measured, and (ii) the Human Protein Atlas of proteins in the retina or hippocampus, to provide a broader reference of human proteins. Using both backgrounds ensures robust enrichment results while mitigating potential biases from incomplete experimental detection. Terms with FDR < 0.05 were considered significant. All normalization, statistical analyses, and data visualizations described above were performed using Python (version 3.13.1).

DAPs were mapped to retinal cell types using single-cell reference data from the Human Cell Atlas Retina v1.0 and visualized via the Cell x Gene Discover platform [39, 55]. DAPs were first plotted to explore their combined expression across all cells in a UMAP embedding of annotated retinal populations. Individual highly changed DAPs were also visualized individually to show their expression patterns across specific cell types.

To provide an expanded network-level view of the retinal proteomic changes, we performed protein–protein interaction network analysis using the STRING database (version 12.0) with all default settings, including a medium confidence interaction score threshold (0.4) and all active interaction sources (co-expression, experimental evidence, curated databases, and text mining). All differentially abundant proteins identified in the AD retina were included as input. The resulting network was partitioned into five clusters using k-means clustering as implemented within the STRING interface. Each cluster was then subjected to functional enrichment analysis using the Molecular Function Gene Ontology category.

## Results

### Retinal AD proteome shows synaptic, cytoskeletal, and mitochondrial changes

**Fig. 2:**
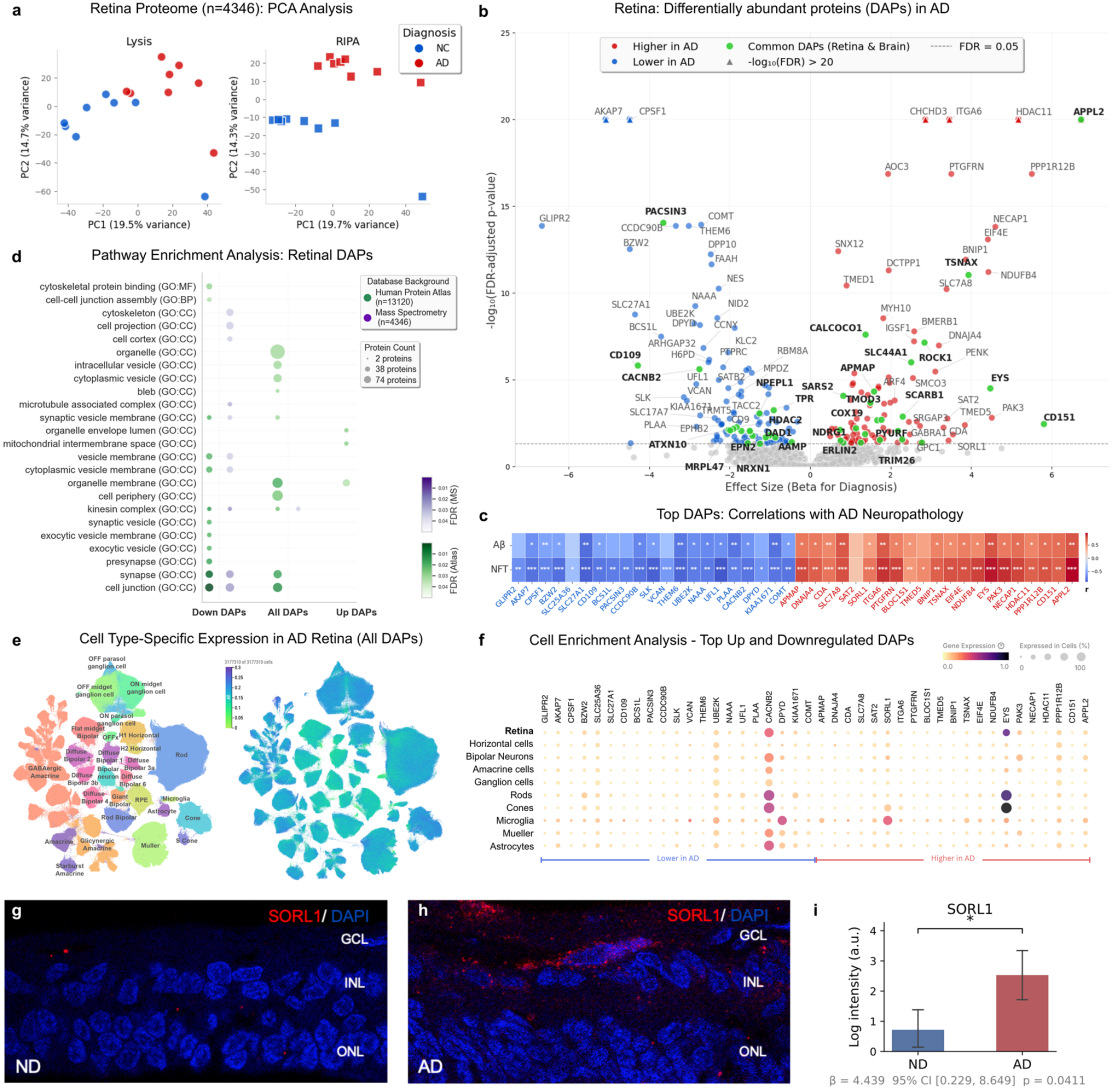
Retinal proteome profiling reveals molecular signatures and suggests cell type-specific alterations in Alzheimer’s disease. **a** Principal component analysis (PCA) of the retinal proteome shows clusters of non-demented controls (NC, blue) and Alzheimer’s disease (AD, red) samples. The left panel represents the Lysis extraction and the right panel the RIPA extraction. **b** Volcano plot displaying 239 differentially abundant proteins (DAPs) in AD retina compared with NC (FDR < 0.05). The x-axis shows the effect size (β coefficient for diagnosis) and the y-axis the statistical significance (− log₁₀ of FDR-adjusted *P *value). Red dots denote proteins elevated in AD, blue dots those reduced in AD, and green dots indicate proteins differentially abundant in both retina and hippocampus. **c** The heatmap shows partial Spearman correlations between top DAPs based on FDR-adjusted *P *values and neuropathological stages (Aβ = amyloid-β plaques; NFT = neurofibrillary tangles), adjusted for age and sex. Blue indicates negative and red positive correlations. Significance is indicated by asterisks (*P < 0.05, **P < 0.01, ***P < 0.001). **d** Top enriched biological terms for downregulated DAPs (left), all DAPs (middle), and upregulated DAPs (right). Dot size reflects the number of proteins per pathway, and color intensity corresponds to statistical significance (− log₁₀ of adjusted *P *value). **e** Cell-type expression patterns of retinal DAPs mapped onto single-cell RNA-sequencing reference data from the Human Cell Atlas Retina v1.0. The left panel shows a UMAP projection of annotated retinal cell populations, and the right panel displays the combined expression of the DAPs across these cell types. **f** Cell-type enrichment analysis of the most significantly upregulated and downregulated DAPs across retinal populations. Dot size indicates the percentage of cells expressing each plotted gene within a given cell type, while color intensity reflects normalized expression relative to all genes shown in the plot (scale bar, top right). **g**–**i** SORL1 immunofluorescence and quantification: representative images from ND (**g**) and AD (**h**) retinas showing SORL1 (red) and DAPI (blue) in retinal layers (GCL = ganglion cell layer; INL = inner nuclear layer; ONL = outer nuclear layer). **i** Quantification shows significantly increased SORL1 levels in AD (*n* = 6) compared with ND (*n* = 6)

After applying quality control to retain only high confidence, consistently detected proteins, 4346 retinal proteins were included in the study. To explore patterns of variation in the retinal proteome, including potential differences associated with disease status, we performed principal component analysis (PCA). Because our dual-buffer extraction protocol was designed to capture proteins from different cellular compartments and/or solubility profiles, we analyzed the Lysis and RIPA fractions separately at this step. In both cases, the PCA revealed clear clustering of AD and NC samples along the first two principal components (Fig. 2a).

In order to identify proteins that differed between AD and NC retinas, we used a linear mixed model adjusted for age and sex. This analysis identified 239 differentially abundant proteins (DAPs; FDR < 0.05), independent of the extraction method (main effect diagnosis) (Fig. 2b) (Supplementary Table 2). Among the DAPs were proteins with well-established relevance to AD and neurodegeneration, including SORL1 and BACE1, both implicated in APP processing, as well as members of the neurexin family of synaptic organizers [44, 47, 76]. Using the same approach, 41 of these retinal DAPs were also detected in the hippocampus.

To further examine how these proteins relate to disease progression, we correlated the top 20 most significantly upregulated and downregulated DAPs with neuropathological stages of amyloid-β and neurofibrillary tangle (NFT) progression. The most highly upregulated proteins, including SORL1, EYS, and APMAP, showed significant positive correlations with neuropathological stages of AD, whereas downregulated proteins, such as CD109, PACSIN3, and VCAN, showed negative correlations (Fig. 2c). LOO analyses supported all significant correlations except the TMED5–NFT association (Supplementary Table 7).

To explore the functions, pathways, and cellular components affected in AD retina, we performed functional enrichment analysis of the DAPs. The enrichment analysis revealed significant alterations across multiple cellular compartments and biological processes. For cellular components (GO:CC), the most prominently enriched terms included organelle-related structures (organelle, organelle membrane), synaptic compartments (synapse, presynapse, synaptic vesicle, synaptic vesicle membrane), cell-cell communication structures (cell junction), and various vesicular transport systems (intracellular vesicle, cytoplasmic vesicle, exocytic vesicle). Notably, mitochondrial compartments (mitochondrial intermembrane space, organelle envelope lumen) displayed enrichment among upregulated DAPs. For biological processes (GO:BP), the analysis identified enrichment in cell-cell junction assembly, consistent with alterations in intercellular communication and structural organization. Molecular Function analysis (GO:MF) revealed enrichment in cytoskeletal protein binding, further supporting disruptions in cellular architecture (FDR < 0.05, Fig. 2d).

To gain insight into the cellular involvement of AD-associated changes in the retina, we mapped our differentially abundant proteins (DAPs) onto single-cell transcriptomic reference data from the Human Cell Atlas Retina v1.0 [39]. When considering all DAPs together, we observed a broad representation across multiple retinal cell types, with a particularly strong signal in ganglion cells, amacrine, and cones (Fig. 2e). Examining the top DAP-associated genes individually revealed that most were not strongly cell-type-specific; however, some were more commonly expressed in microglia, with both increases and decreases observed in AD. Among these, DPYD and SORL1 stood out as more prominent, displaying relatively high expression in microglia. Other genes, such as APMAP, VCAN, and TSNAX, were also more detected in microglia than in other cell types, though at lower levels and restricted to a percentage of cells. Notably, EYS, which was increased in AD, exhibited pronounced expression in rods and cones (Fig. 2f) (Supplementary Fig. 1).

Among the retinal DAPs, SORL1 was selected for orthogonal validation by immunohistochemistry, given its consistent differential abundance across both extraction buffers and its established relevance to both Alzheimer’s disease and retinal pathology [25, 44] (Fig. 2g–i).

### Sequential extraction reveals buffer-dependent AD-associated changes in APP processing

To extend our findings beyond buffer-independent effects, we next examined how extraction conditions influenced the detection of AD-related changes. Because Lysis and RIPA solubilize partly overlapping but distinct protein pools, the Diagnosis × Buffer interaction in our model allowed us to identify alterations whose magnitude differed between buffers, as well as changes detectable under one extraction condition only, potentially reflecting differences in protein solubility, subcellular localization, or incorporation into tightly bound complexes.

**Fig. 3:**
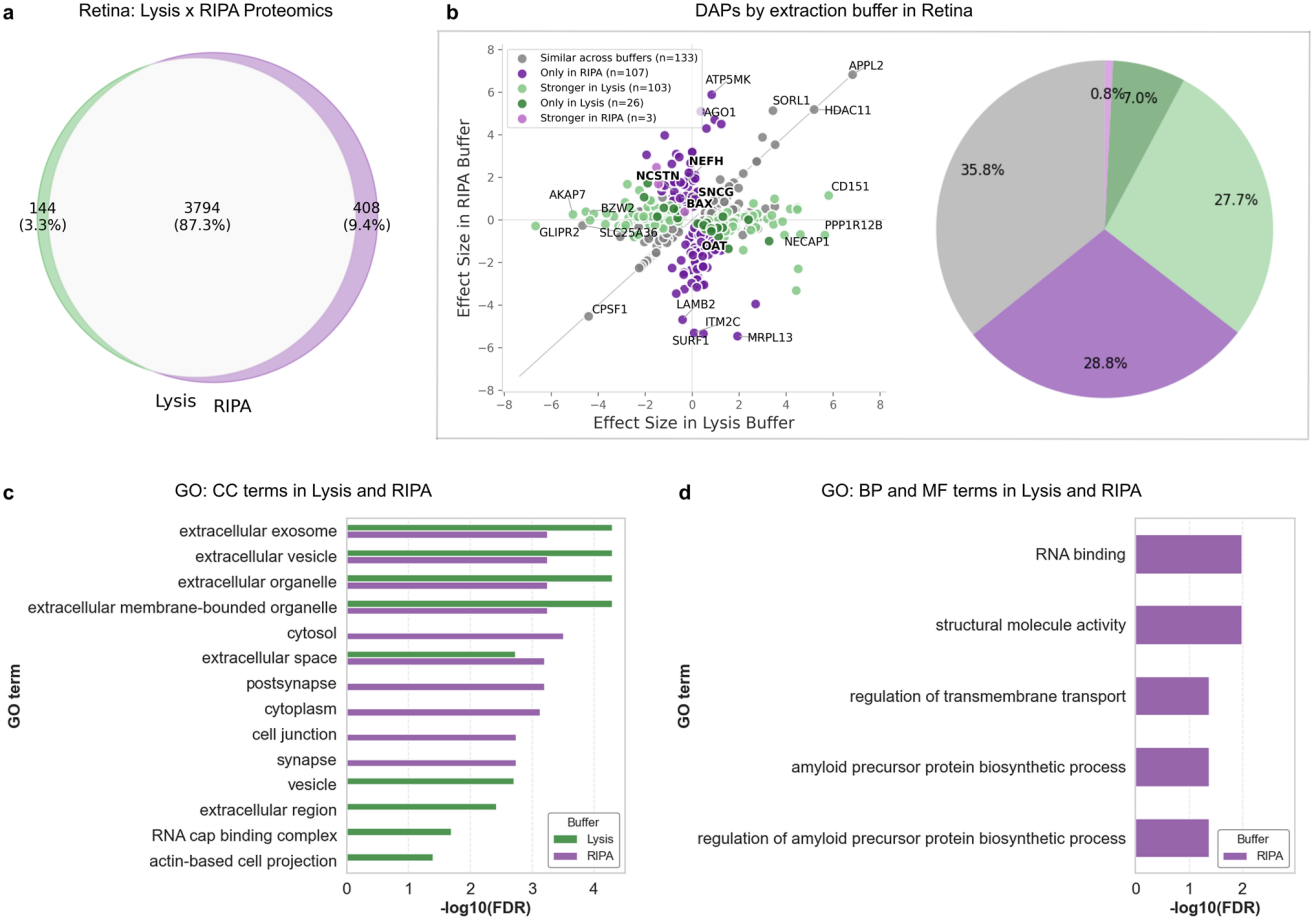
Comparative proteomic analysis of Lysis versus RIPA extraction reveals buffer-specific protein recovery and AD-associated changes in APP processing. **a** Venn diagram showing overlap of proteins identified by Lysis and RIPA buffers in retina. **b** Scatter plot (left) comparing effect sizes of differentially abundant proteins (DAPs) between extraction buffers, where x-axis represents the effect size in Lysis buffer and y-axis represents the effect size in RIPA buffer. Proteins are color-coded by their extraction profile. Pie chart (right) shows the proportional distribution of DAP categories (*n* = 372 total). **c** Gene Ontology Cellular Component (GO:CC) enrichment analysis showing terms significantly enriched in proteins extracted by RIPA (orange) or Lysis (green) buffers. **d** Gene Ontology Biological Process (GO:BP) and Molecular Function (GO:MF) enrichment analysis for buffer-specific protein profiles. Statistical significance expressed as -log10 (q-value)

Proteomic comparison of the two fractions identified 4346 retinal proteins in total: 3,794 (87.3%) were shared across both buffers, while 144 (3.3%) and 408 (9.4%) were unique to Lysis and RIPA, respectively (Fig. 3a). Among the DAPs with a main effect of diagnosis, indicating consistent AD-related changes across buffers, several also showed a significant Diagnosis × Buffer interaction, reflecting a stronger effect in one extraction method. Additional proteins exhibited interaction effects only, indicating that their AD-associated changes were exclusively captured in a single buffer (Fig. 3b) (Supplementary Table 2).

Among the total 372 DAPs identified in the retina, 239 showed consistent disease-related changes across buffers. Of these, 133 (35.8%) exhibited similar abundance in both buffers, 103 (27.7%) were more abundant in Lysis, and 3 (0.8%) were more abundant in RIPA. The remaining 133 DAPs displayed buffer-dependent effects only, with 107 (28.8%) detected exclusively in RIPA and 26 (7.0%) exclusively in Lysis. Altogether, buffer-dependent proteins, those presenting stronger or exclusive extraction patterns, accounted for the majority (64.2%) of disease-associated proteins identified (Fig. 2b). Notably, several proteins associated with critical pathological processes were only found to be different in RIPA buffer, including NCSTN, a component of the gamma-secretase complex that processes APP[45]; BAX, a central mediator of retinal ganglion cell death [42]; NEFH and SNCG, previously associated with glaucomatous damage; and OAT, deficiency of which causes gyrate atrophy of the choroid and retina [70].

Gene Ontology enrichment analysis revealed distinct Cellular Component profiles between the two extraction buffers. Lysis, using mild conditions to extract soluble and loosely associated proteins, was enriched for extracellular vesicles, exosomes, and membrane-bounded organelles, whereas RIPA, using detergents to extract proteins from compartments resistant to mild Lysis and those in protein complexes, preferentially captured proteins from the cytosol, postsynaptic regions, and cell junctions. Both buffers captured extracellular and organelle-associated proteins, but with different efficiencies (Fig. 3c). Functional enrichment analysis further showed that RIPA-extracted proteins were overrepresented in biological processes related to transmembrane transport and amyloid precursor protein biosynthesis, and in molecular functions such as RNA binding and structural molecule activity (Fig. 3d).

To explore the network-level organization of all retinal DAPs, regardless of extraction condition, protein–protein interaction analysis was performed using STRING, and k-means clustering identified five co-functional modules (Supplementary Fig. 2a–e). The enriched molecular functions per cluster included: cytoskeletal protein binding (Cluster 1), integrin and amyloid-beta binding (Cluster 2), mRNA and RNA binding (Cluster 3), and oxidoreductase activity (Cluster 4).

### Retina and hippocampus show strong proteomic similarity, and 64 share DAPs in AD

**Fig. 4:**
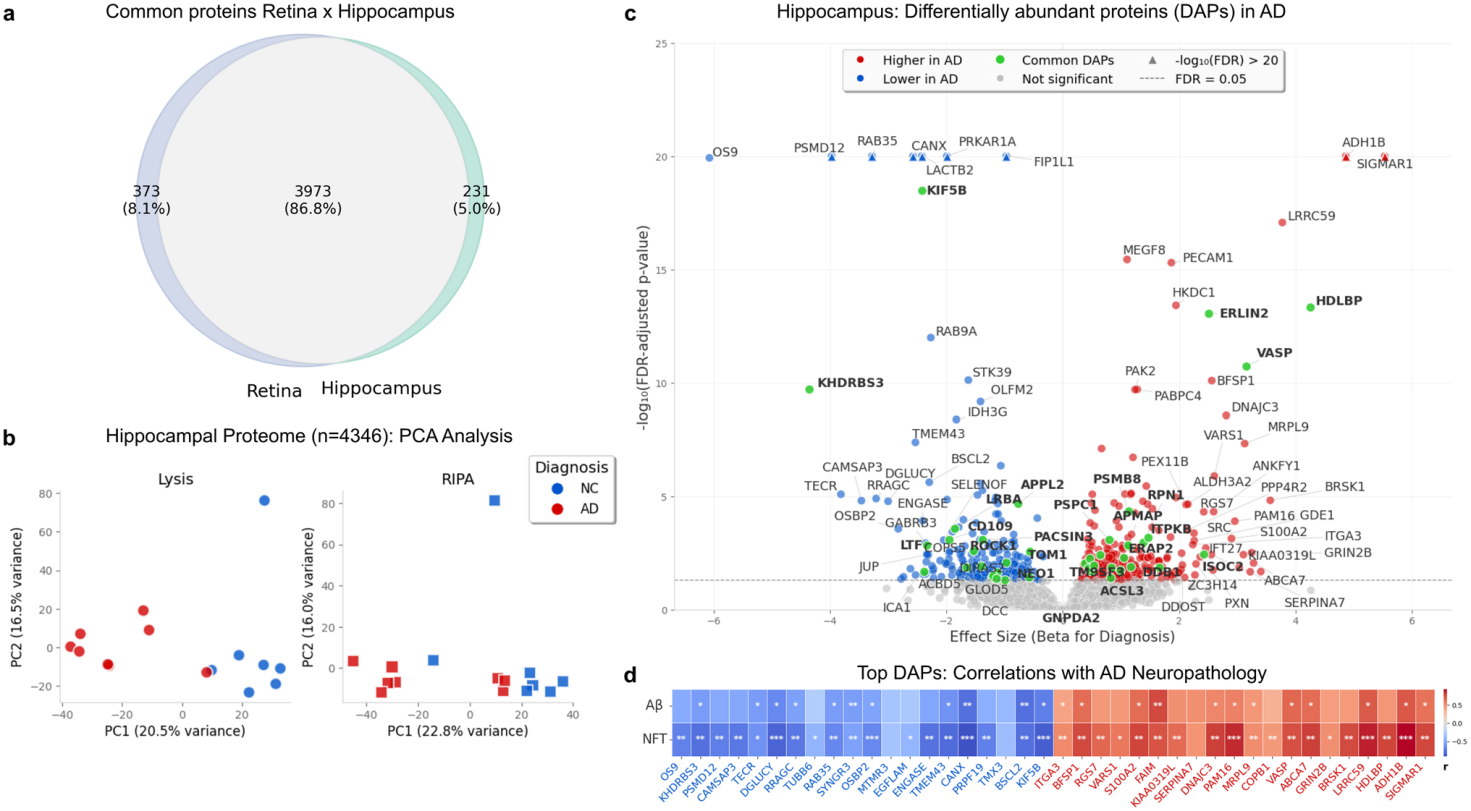
The hippocampal proteome shows extensive overlap with the retina and shared differentially abundant proteins (DAPs). **a** Venn diagram illustrating the overlap between proteins identified in the retina (purple) and hippocampus (green). **b** Principal component analysis (PCA) of the hippocampal proteome reveals a clustering pattern separating non-demented controls (NC, blue) and Alzheimer’s disease (AD, red) samples, particularly in the Lysis fraction. The left and right panels represent the Lysis and RIPA extractions, respectively. **c** Volcano plot displaying 484 DAPs in the AD hippocampus compared with NC (FDR < 0.05). The x-axis represents the effect size (β coefficient for diagnosis) and the y-axis the statistical significance (− log₁₀ adjusted *P *value). Red dots denote proteins upregulated in AD, blue dots those downregulated in AD, and green dots indicate proteins differentially abundant in both retina and hippocampus. **d** The heatmap shows partial Spearman correlations between top DAPs and neuropathological stages (Aβ = amyloid-β plaques; NFT = neurofibrillary tangles), adjusted for age and sex. Blue indicates negative and red positive correlations. Significance is indicated by asterisks (*P < 0.05, **P< 0.01, ***P < 0.001)

In the hippocampal CA1 region, 4204 proteins were identified using the same quality control criteria applied to the retina, of which 3973 (87%) were also detected in the retina, highlighting a substantial proteomic overlap between the two tissues (Fig. 4a).

To assess overall proteomic variation, we performed principal component analysis (PCA) of the hippocampal dataset. A clustering pattern separating Alzheimer’s disease (AD) and non-demented control (NC) samples was observed along the first two principal components, particularly in the Lysis fraction (Fig. 4b).

Differential abundance analysis using a linear mixed model adjusted for age and sex identified 484 proteins with significant differences between AD and NC, regardless of extraction method (FDR < 0.05; Fig. 4c). To evaluate the relationship between protein abundance changes and AD pathology severity, we examined partial Spearman correlations between the top DAPs and stages of neuropathological severity, amyloid-β plaques and NFT. Most upregulated proteins correlated positively with neuropathological burden, while downregulated proteins correlated negatively with disease stage (Fig. 4d). Several proteins previously linked to AD in human hippocampal proteomics were also observed here [28, 58]. For instance, synapse-related proteins, including SYP, YWHAG, DLAT, and PDHB, were downregulated, whereas AQP4, GJA1, and HSPB1, associated with inflammatory or stress-related processes, were upregulated. Notably, APOE levels were also increased in AD in our data.

To evaluate whether buffer-dependent extraction patterns observed in the retina were also present in the brain, we performed a similar analysis in the hippocampal CA1 region. A total of 696 differentially abundant proteins (DAPs) were identified in the hippocampus when considering buffer effects (Supplementary Table 3). Among these, 64 DAPs were also detected in the retina. Dividing the DAPs by extraction method, 363 (52.2%) exhibited similar changes across both buffers, for 118 (17.0%) the changes were stronger in Lysis, and 3 (0.4%) were stronger in RIPA. The remaining 212 DAPs presented buffer-dependent effects only, with 145 (20.8%) showing changes exclusively in RIPA and 67 (9.6%) exclusively in Lysis (Supplementary Fig. 3).

### Retina and brain show overlapping pathway changes and correlated AD-associated proteins

To assess whether AD-associated proteomic changes in the retina reflect those occurring in the brain, we compared all 372 retinal DAPs (including extraction-dependent ones) with the 696 DAPs identified in the hippocampus. By considering the complete set of disease-associated proteins regardless of extraction method, we aimed to capture the full spectrum of retinal proteomic changes and their relationship to brain pathology. We first examined overlap at the individual protein level to identify shared disease-associated proteins between tissues.

**Fig. 5:**
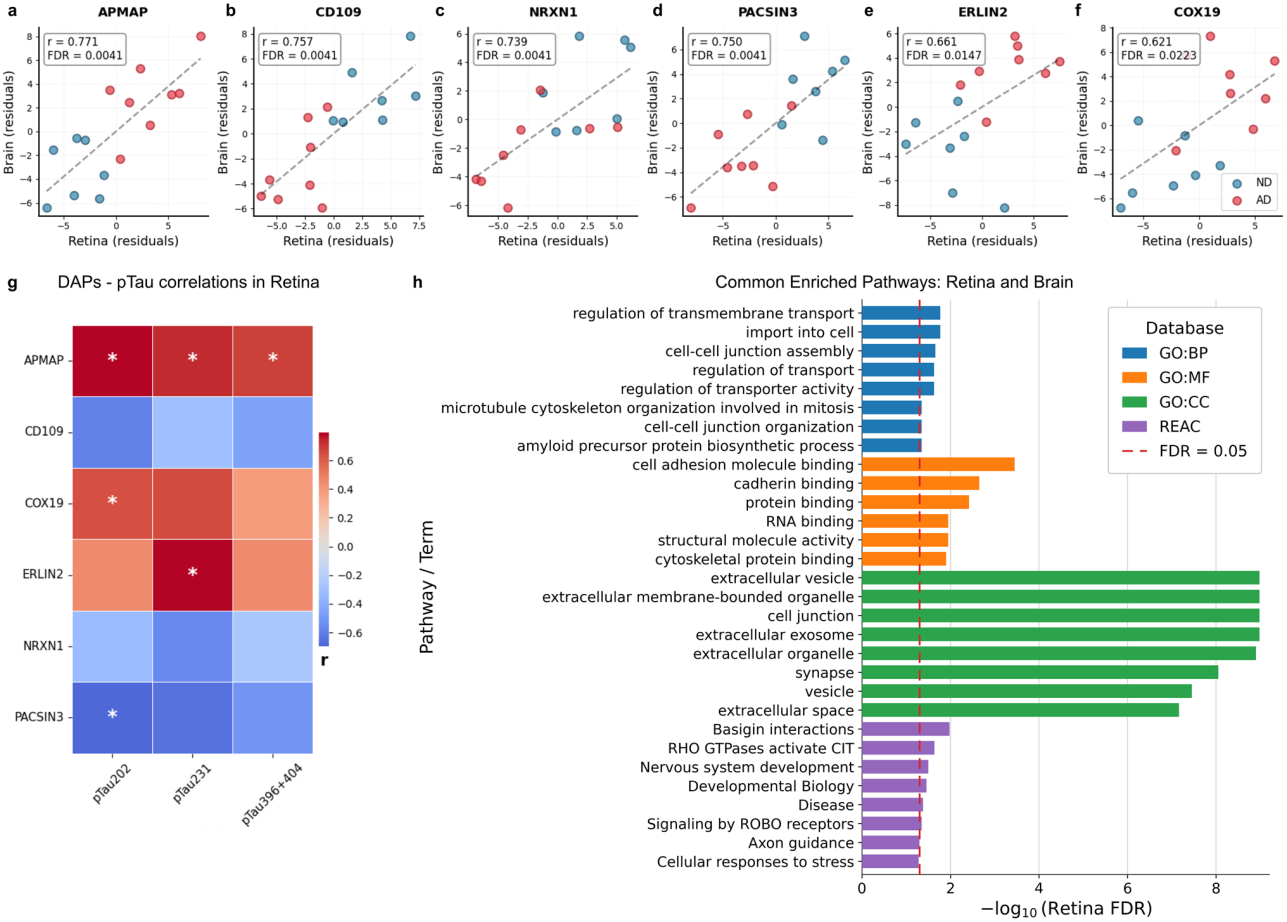
Retina and brain show common enriched pathways and proteins with strong cross-tissue correlations. **a**–**f** Scatter plots demonstrating partial Spearman correlations between retinal and hippocampal protein abundance (residuals adjusted for age and sex) for six proteins with robust cross-tissue associations: APMAP, CD109, NRXN1, PACSIN3, ERLIN2, and COX19. Each point represents an individual sample (ND in blue, AD in red). Dashed lines indicate linear regression fits. Correlation coefficients (r) and FDR-corrected *P *values are shown in each panel. **g** Heatmap of Spearman correlations between these six proteins and retinal tau phosphorylation sites (pThr12, pSer199, pThr231, pSer396/404). Red indicates positive correlations; blue indicates negative correlations. Significance is indicated by asterisks (*FDR < 0.05, **FDR < 0.01). **h** Gene Ontology enrichment analysis showing pathways/terms commonly enriched in retina and hippocampus in AD (FDR < 0.05), based on all proteins analyzed. Bar colors indicate pathway categories: Biological Process (blue), Molecular Function (orange), Cellular Component (green), and Reactome pathways (purple)

At the individual protein level, 29 of the 64 DAPs shared by both tissues exhibited changes in the same direction. Among proteins upregulated in both tissues, three showed correlated values between retina and brain: APMAP, ERLIN2, and COX19. Among proteins downregulated in both tissues, three correlated: CD109, NRXN1 and PACSIN3 (Fig. 5 a–f and Supplementary Table 4). LOO analyses supported all retina–brain correlations, which remained significant across all iterations (Supplementary Table 7). These associations were attenuated and lost significance after adjustment for diagnosis. Immunofluorescence microscopy of APMAP and PACSIN3, which showed more prominent changes in the Lysis buffer by mass spectrometry, did not reveal statistically significant differences in stained area between AD and ND retinal samples (Supplementary Fig. 4).

Given the relevance of tau pathology in AD, we next examined whether retinal levels of these six proteins were associated with retinal phosphorylated tau (p-tau) at phosphorylation sites (pTau202, pTau231, pTau396 + 404), previously reported to be affected in the presence of AD pathology [60]. Partial Spearman correlation analysis adjusted for age and sex revealed significant positive correlations of APMAP with all three phosphorylation sites, a positive correlation of COX19 with pTau202, and a positive correlation of ERLIN2 with pTau231. PACSIN3 showed a significant negative correlation with pTau202 (Fig. 4g). After additionally adjusting for diagnosis, only ERLIN2, APMAP, and COX19 retained nominally significant positive correlations with pTau231 (*p* < 0.05), though none survived correction for multiple comparisons.

Beyond individual protein overlap, we performed functional enrichment analysis on all differentially abundant proteins from both tissues. Analyses included the combined set of proteins, upregulated and downregulated proteins separately, and buffer-specific profiles for Lysis and RIPA. This approach allowed us to determine whether retina and hippocampus share convergent biological processes and pathways despite potential differences in specific protein identities. Gene Ontology enrichment analysis revealed several shared pathways between retina and brain DAPs (Fig. 5h). Biological processes included regulation of transmembrane transport, import into cell, cell–cell junction assembly, and amyloid precursor protein biosynthetic process. Molecular Function terms encompassed cell adhesion molecule binding, cadherin binding, and cytoskeletal protein binding. Cellular Component analysis showed enrichment in extracellular exosome, extracellular organelle, cell junction, and synaptic compartments. Reactome pathway analysis highlighted Basigin interactions, RHO GTPases activate CIT, axon guidance, nervous system development, and metabolism of amino acids and derivatives as shared pathways.

## Discussion

Our proteomic analysis reveals molecular alterations in the AD retina that suggest involvement of multiple pathological pathways. The separation of AD and control samples in the principal component analysis indicates the disease seems to have a profound effect on the retinal (and hippocampal) proteome, supported by 372 proteins showing differential abundance between AD retinas and controls. The substantial proteomic overlap between retina and hippocampus (87% of total proteins) provides a foundation for comparison, with 64 proteins showing disease-associated changes in both tissues. Among these 29 DAPs exhibited changes in the same direction, with some exhibiting strong cross-tissue correlations. Pathway enrichment analyses of differentially abundant proteins revealed several altered pathways that we discuss under five major categories: (1) synaptic and vesicular processes; (2) cytoskeleton and intracellular transport; (3) APP processing and membrane signaling; (4) mitochondrial function and cellular stress; and (5) cell–cell junctions, adhesion, and barrier integrity.

Synaptic and vesicular processes were the most prominent category altered, with enrichment of terms related to synaptic organization (pre- and post-synapse, glutamatergic synapse) and vesicle recycling machinery in both tissues. Within this category, several synaptic organizers, including NRXN1, NRXN3, NECAB2, and CACNB2, were significantly reduced in AD retina. These changes align with robust evidence linking early synaptic protein loss in the brain to tau pathology and cognitive decline [69, 74], where synaptic alterations are among the strongest predictors of cognitive impairment [4, 50]. Our findings suggest that AD-related synaptic remodeling is extended to retinal neurons. Previous studies corroborate this concept: rodent models indicate early retinal synaptic changes associated with Aβ and tau, including synapse loss in the inner plexiform layer and altered neurotransmission [14, 40], and suggest that brain-derived Aβ can reach the retina via the optic nerve, causing synaptic dysfunction [13]. Whether synaptic changes in the human retina occur independently of, or precede, local Aβ and tau deposition is an open question.

Cytoskeleton and intracellular transport was another strongly altered category, suggesting widespread disruption of structural and trafficking networks in both retina and brain. Enriched terms included microtubule organization, actin-based projections, and motor protein complexes such as kinesins and dyneins [82]. In brain tissue, hyperphosphorylated tau destabilizes microtubules, impairs axonal transport, and contributes to synaptic dysfunction and neurodegeneration [31, 52, 57]. Similar mechanisms appear to operate in the retina as tau accumulation and phosphorylation have been observed in human AD retinas by multiple groups [16, 18, 24, 60, 75]. Experimental studies further indicate that amyloid-β can disrupt axonal transport and compromise cytoskeletal stability in both the brain and retina [20]. Together, these findings suggest that cytoskeletal and transport deficits represent a systemic vulnerability in AD, affecting both central and retinal circuits and potentially contributing to early visual dysfunction before overt neuronal loss.

APP processing is a central pathway in Alzheimer’s disease, as its proteolytic cleavage determines whether non-amyloidogenic fragments or amyloidogenic Aβ peptides are produced. In the brain, dysregulation of this pathway drives Aβ accumulation, plaque formation, and downstream synaptic and signaling disturbances [10, 71]. Whether analogous changes occur in the retina has been debated, with some studies reporting Aβ deposition and APP-related changes in retinal tissue [33, 38, 61] and others finding no significant differences [18]. Our retinal proteomic data provide evidence for APP-related molecular remodeling in the eye, with enrichment of terms including “amyloid precursor protein biosynthetic process” and “regulation of APP biosynthetic process,” supported by changes in key proteins such as SORL1, BACE1, and APBB1. Importantly, these pathway changes were detected exclusively in the RIPA-soluble fraction, suggesting that APP/Aβ-related alterations may be compartment- or solubility-dependent and potentially only detectable through sequential extraction approaches. Taken together, while discrepancies exist, our results suggest the retina exhibits at least some amyloid-related molecular remodeling.

Mitochondrial dysfunction and cellular stress are recognized features of Alzheimer’s disease. In brain tissue, impaired oxidative phosphorylation, disrupted mitochondrial dynamics, and increased reactive oxygen species contribute to synaptic failure and neuronal degeneration [2, 77]. Our retinal proteomic analysis revealed enrichment of terms such as ‘mitochondrion,’ ‘mitochondrial intermembrane space,’ and stress-related pathways, including ‘cellular responses to stress’ and ‘lysosome,’ suggesting that similar bioenergetic challenges may occur in retinal tissue.

Disruption of cell–cell junctions and adhesion complexes is increasingly recognized as another contributor to Alzheimer’s disease pathology. In the brain, the integrity of the blood–brain barrier (BBB) depends on tight junctions (claudins, occludin, ZO-1), adherens junctions (VE-cadherin), and gap junctions formed by connexins. Loss or disorganization of these junctional proteins leads to increased vascular permeability, neuroinflammation, and impaired neuronal signaling [68, 80]. Our retinal proteomic analysis revealed enrichment of pathways such as ‘cell–cell junction organization,’ ‘apical junction complex,’ ‘focal adhesion,’ and ‘cadherin binding,’ suggesting potential effects on the blood–retina barrier (BRB). Experimental models have suggested that retinal vascular amyloid deposition correlates with tight junction loss and BRB breakdown, paralleling cerebral amyloid angiopathy [64, 65]. Whether similar mechanisms contribute to retinal pathology in human AD warrants further investigation.

To date, only one prior proteomic study has examined retinal changes in AD. The study, conducted by Koronyo et al. [34], analyzed the proteome of 6 AD and 6 control retinas, but the analytical and methodological approaches differed substantially from ours. Koronyo et al. used functional annotation to provide a descriptive overview of affected processes, whereas our study applies formal statistical pathway enrichment, allowing identification of coordinated molecular networks. Moreover, different retinal regions were analyzed in the two studies, and while Koronyo et al. used a single-buffer extraction, we employed a dual-extraction strategy to capture proteins of differing solubility. Finally, our statistical framework differed substantially from the previous study. Given these differences in study design and methodology, direct protein-level comparisons between the two studies should be interpreted with caution, while broader biological processes are more comparable. Koronyo et al. reported inflammatory, neurodegenerative, mitochondrial, and photoreceptor-related alterations, findings that our data broadly corroborate and extend. Another important difference between the two studies concerns how retinal and brain proteomes were compared. In the previous study, the retinal proteome was compared with brain data generated from an independent donor cohort [34]. In contrast, our study uses paired same-donor retina–brain samples, reducing inter-individual variability and enabling direct cross-tissue comparison. We therefore have the possibility to directly compare retinal and brain proteomic changes within the same individuals.

Our analysis revealed a substantial proteomic overlap between retina and hippocampus, with 87% of proteins shared across both tissues. Despite this extensive overlap, disease-associated changes were not uniform: 64 differentially abundant proteins (DAPs) were common to both tissues, yet only 29 changed in the same direction. Importantly, although individual proteins did not consistently follow the same trend, the broader pathways were altered similarly in both tissues, indicating downregulation of synaptic and cytoskeletal processes and upregulation of mitochondrial compartments and stress-related pathways, for example. This divergence at the protein level, despite pathway-level similarity, could reflect differences in disease stage, local adaptive responses, or tissue-specific vulnerability. For example, SORL1, a protein involved in APP processing, displayed increased abundance in the retina in our data, which may represent an adaptive response, whereas downregulation of this protein has been associated with AD in brain tissue [44]. Such bidirectional regulation is well documented in AD, where early compensatory responses such as increased synaptic plasticity and transient neuroinflammatory activity that initially promote clearance of toxic species eventually fail, leading to progressive neurodegeneration [27, 48]. The increased abundance of SORL1 in the AD retina deserves special attention, as this protein has been implicated in retinal pathologies beyond its well-established role in Alzheimer’s disease. SORL1, also known as SORLA and LR11, has been linked to age-related retinal changes, with SORL1 knockout mice displaying abnormal retina morphology in late-adult phenotyping, and STRING analysis suggesting that SORL1 forms a network with age-related macular degeneration (AMD) GWAS genes [23]. Moreover, elevated vitreous levels of soluble SORL1 have been found in idiopathic epiretinal membrane [25] and diabetic retinopathy [67]. These observations suggest that SORL1 dysregulation may affect retinal homeostasis across multiple disease contexts, and that its upregulation in the AD retina could reflect shared molecular mechanisms between Alzheimer’s disease and other disorders affecting the retina.

Among the common retina–brain DAPs, six showed significant cross-tissue correlations within individuals: APMAP, ERLIN2, and COX19 (upregulated in both tissues) and CD109, NRXN1, and PACSIN3 (downregulated in both). These within-subject correlations indicate that, for a subset of proteins, the magnitude of retinal change tracks the corresponding brain change, further supporting the concept that the retina reflects aspects of cerebral molecular pathology. Importantly, these proteins should be regarded as indicators of shared molecular alterations between retina and brain, and their interpretation as candidate biomarkers should be made with caution, particularly given that our sequential dual-extraction approach was optimized for mechanistic characterization rather than biomarker discovery.

Mapping all DAPs to single-cell retinal data revealed a broad distribution across cell types, with prominent representation in ganglion cells, consistent with previous reports of ganglion cell loss in AD [16, 35, 46]. DAPs were also overrepresented in amacrine cells, which are interneurons, and intriguingly, in cones, a photoreceptor type unique to the retina. While overall enrichment patterns lacked strong cell-type specificity, some highly altered proteins displayed preferential expression in particular cell types. For example, SORL1 and APMAP were primarily enriched in microglia, supporting evidence of microglial involvement in the AD retina [34, 49, 78]. Although we hypothesized dysregulation of inflammatory pathways, formal enrichment analysis did not identify these at the pathway level. This may reflect the limited statistical power of the current sample size, the possibility that inflammatory changes are stage-specific and thus less prominent in end-stage postmortem tissue, or that such changes in the retina are more subtle or cell-type restricted than in the brain, and may require targeted analyses or single-cell approaches to be fully resolved. Notably, EYS (EGF-like photoreceptor maintenance factor), predominantly expressed in photoreceptors and a major gene for rod-cone dystrophies [6, 29], was among the most upregulated proteins in AD retinas. While the previous proteomics study reported downregulation of some photoreceptor-related proteins [34], the increase in EYS observed here may reflect differences in the retinal regions analyzed (superior versus temporal) or differential responses among photoreceptor protein subclasses, while nevertheless supporting the common observation of photoreceptor involvement. Cell-type mapping further showed enrichment of differentially abundant proteins in cones, suggesting that photoreceptor-related changes extend beyond ganglion cells and inner retinal neurons. Although bulk proteomics has inherent limitations for cell-type attribution and this enrichment may partly reflect shifts in relative retinal composition, the photoreceptor-specific expression of EYS indicates that AD-related molecular alterations may also affect outer retina. This warrants further investigation, as photoreceptors represent a retina-specific cell type that may reveal unique aspects of AD neurodegeneration in this tissue.

### Limitations

This study provides valuable insight into the molecular changes occurring in the retina in AD and how they correlate with alterations in the brain of the same individuals; however, several limitations should be considered. First, the use of postmortem tissue from individuals with established dementia reflects predominantly end-stage disease and therefore may not capture earlier or more dynamic molecular changes that occur during AD initiation or progression. The sample size was small, which reduced statistical power for detecting moderate or subtle effect sizes and limited the ability to perform subgroup or stage-specific analyses. Even so, the study was sufficiently powered to identify robust disease-associated differences, as reflected by the separation of diagnostic groups in principal component analysis. Although postmortem delay was consistent between diagnostic groups, the use of postmortem tissue also introduces factors, such as tissue handling and protein degradation, that may not fully reflect in vivo conditions. In addition, analyses were restricted to a single retinal region, which may not capture spatial heterogeneity across the retina. Cohort composition also represents a potential source of residual confounding. Although sex was included as a covariate in all statistical models, the female predominance in the AD group cannot be fully excluded as a contributing factor, particularly given the well-documented sex differences in AD biology and molecular pathology. Replication in larger, sex-balanced cohorts will be important to confirm the specificity of these findings. Finally, cell-type enrichment was inferred from transcriptomic reference datasets, which do not always correspond to protein-level expression.

## Conclusion

By providing a systems-level proteomic characterization of the AD retina, this study advances our understanding of the molecular basis underlying the structural changes reported in in vivo imaging and histological studies. Using a paired retina–brain design, an approach rarely explored in retinal AD research, we show that the AD retina undergoes molecular alterations that parallel cerebral pathology. These include pathway-level changes in synaptic function, cytoskeletal organization, and APP processing, none of which have been described in the retina before. Retinal proteomic changes were also associated with measures of neuropathological burden, suggesting a relationship between retinal molecular alterations and disease severity. The sequential extraction approach provides additional insight into protein solubility states and implies that APP/Aβ-related changes may be solubility- or compartment-dependent, and some evidence points to retina-specific features in AD, such as altered photoreceptor proteins. While validation in larger cohorts is needed, these findings establish a molecular foundation for understanding retinal AD pathology and support the retina as an accessible window for monitoring neurodegeneration in Alzheimer’s disease.

## Supplementary Information

Below is the link to the electronic supplementary material.

Supplementary file1 (DOCX 3973 KB)

Supplementary file2 (XLSX 1550 KB)

## Data Availability

The mass spectrometry proteomics data have been deposited to the ProteomeXchange Consortium via the PRIDE [62] partner repository with the dataset identifier PXD073336. Data are pseudonymized and comply with GDPR regulations and the ethical approval granted by the Medical Ethical Committee of the VU Medical Center, Amsterdam. The processed data generated during the analysis, supporting the results presented in this study, are available within the main manuscript and the Supplementary Information files.

## Abbreviations

ACN: Acetonitrile
AD: Alzheimer’s disease
AGC: Automatic gain control
Aβ: Amyloid-beta
APP: Amyloid precursor protein
APOE4: Apolipoprotein E ε4 allele
BORC: Biogenesis of lysosome-related organelles complex
BP: Biological Process (Gene Ontology)
CC: Cellular Component (Gene Ontology)
CSF: Cerebrospinal fluid
DAPs: Differentially abundant proteins
DDA: Data-dependent acquisition
DTT: Dithiothreitol
EDTA: Ethylenediaminetetraacetic acid
FA: Formic acid
FDR: False discovery rate
GO: Gene Ontology
HCD: Higher-energy collisional dissociation
HDAC: Histone deacetylase
LC–MS/MS: Liquid chromatography–tandem mass spectrometry
LOO: Leave-one-out cross-validation
MF: Molecular Function (Gene Ontology)
MNAR: Missing-not-at-random
MS1/MS2: Mass spectrometry level 1 and 2 scans
NFT: Neurofibrillary tangle
NC: Non-demented control
OCT: Optical coherence tomography
p-tau: Phosphorylated tau
PCA: Principal component analysis
PRIDE: Proteomics identifications database
RIPA: Radioimmunoprecipitation assay buffer
RNA: Ribonucleic acid
TFA: Trifluoroacetic acid
UHPLC: Ultra-high-performance liquid chromatography

## References

[1] (2025) 2025 Alzheimer’s disease facts and figures. Alzheimer’s & Dementia 21: e70235 10.1002/alz.70235

[2] Adav SS, Park JE, Sze SK (2019) Quantitative profiling brain proteomes revealed mitochondrial dysfunction in Alzheimer’s disease. Mol Brain 12:8. 10.1186/s13041-019-0430-y 30691479 10.1186/s13041-019-0430-y PMC6350377

[3] Alvite-Pineiro T, Lopez-Lopez M, Regueiro U, Pias-Peleteiro JM, Sobrino T, Lema I (2025) Visual function in Alzheimer’s disease: current understanding and potential mechanisms behind visual impairment. J Clin Med. 10.3390/jcm14175963 40943721 10.3390/jcm14175963 PMC12429424

[4] Ao J, Picard C, Auld D, Zetterberg H, Brinkmalm A, Blennow K et al (2025) Novel synaptic markers predict early tau pathology and cognitive deficit in an asymptomatic population at risk of Alzheimer’s disease. Mol Psychiatry 30:2810–2820. 10.1038/s41380-024-02884-z 39827219 10.1038/s41380-024-02884-z

[5] Ardanaz CG, Ramirez MJ, Solas M (2022) Brain metabolic alterations in Alzheimer’s disease. Int J Mol Sci. 10.3390/ijms23073785 35409145 10.3390/ijms23073785 PMC8998942

[6] Audo I, Sahel JA, Mohand-Said S, Lancelot ME, Antonio A, Moskova-Doumanova V et al (2010) EYS is a major gene for rod-cone dystrophies in France. Hum Mutat 31:E1406-1435. 10.1002/humu.21249 20333770 10.1002/humu.21249

[7] Banna HU, Slayo M, Armitage JA, Del Rosal B, Vocale L, Spencer SJ (2024) Imaging the eye as a window to brain health: frontier approaches and future directions. J Neuroinflammation 21:309. 10.1186/s12974-024-03304-3 39614308 10.1186/s12974-024-03304-3 PMC11606158

[8] Bano D, Ehninger D, Bagetta G (2023) Decoding metabolic signatures in Alzheimer’s disease: a mitochondrial perspective. Cell Death Discov 9:432. 10.1038/s41420-023-01732-3 38040687 10.1038/s41420-023-01732-3 PMC10692234

[9] Blanks JC, Torigoe Y, Hinton DR, Blanks RH (1996) Retinal pathology in Alzheimer’s disease. I. Ganglion cell loss in foveal/parafoveal retina. Neurobiol Aging 17:377–384. 10.1016/0197-4580(96)00010-3 8725899 10.1016/0197-4580(96)00010-3

[10] Bossy-Wetzel E, Schwarzenbacher R, Lipton SA (2004) Molecular pathways to neurodegeneration. Nat Med 10(Suppl):S2-9. 10.1038/nm1067 15272266 10.1038/nm1067

[11] Braak H, Braak E (1991) Neuropathological stageing of Alzheimer-related changes. Acta Neuropathol 82:239–259. 10.1007/BF00308809 1759558 10.1007/BF00308809

[12] Busche MA, Hyman BT (2020) Synergy between amyloid-beta and tau in Alzheimer’s disease. Nat Neurosci 23:1183–1193. 10.1038/s41593-020-0687-6 32778792 10.1038/s41593-020-0687-6 PMC11831977

[13] Cao Q, Yang S, Wang X, Sun H, Chen W, Wang Y et al (2024) Transport of beta-amyloid from brain to eye causes retinal degeneration in Alzheimer’s disease. J Exp Med. 10.1084/jem.20240386 39316084 10.1084/jem.20240386 PMC11448872

[14] Chang LY, Ardiles AO, Tapia-Rojas C, Araya J, Inestrosa NC, Palacios AG et al (2020) Evidence of synaptic and neurochemical remodeling in the retina of aging degus. Front Neurosci 14:161. 10.3389/fnins.2020.00161 32256305 10.3389/fnins.2020.00161 PMC7095275

[15] Curcio CA, Drucker DN (1993) Retinal ganglion cells in Alzheimer’s disease and aging. Ann Neurol 33:248–257. 10.1002/ana.410330305 8498808 10.1002/ana.410330305

[16] Davis MR, Robinson E, Koronyo Y, Salobrar-Garcia E, Rentsendorj A, Gaire BP et al (2025) Retinal ganglion cell vulnerability to pathogenic tau in Alzheimer’s disease. Acta Neuropathol Commun 13:31. 10.1186/s40478-025-01935-y 39955563 10.1186/s40478-025-01935-y PMC11829413

[17] De Strooper B, Karran E (2016) The cellular phase of Alzheimer’s disease. Cell 164:603–615. 10.1016/j.cell.2015.12.056 26871627 10.1016/j.cell.2015.12.056

[18] den Haan J, Morrema THJ, Verbraak FD, de Boer JF, Scheltens P, Rozemuller AJ et al (2018) Amyloid-beta and phosphorylated tau in post-mortem Alzheimer’s disease retinas. Acta Neuropathol Commun 6:147. 10.1186/s40478-018-0650-x 30593285 10.1186/s40478-018-0650-x PMC6309096

[19] den Haan J, Verbraak FD, Visser PJ, Bouwman FH (2017) Retinal thickness in Alzheimer’s disease: a systematic review and meta-analysis. Alzheimers Dement Diagn Assess Dis Monit 6:162–170. 10.1016/j.dadm.2016.12.014 10.1016/j.dadm.2016.12.014 PMC5328759 28275698

[20] Deng L, Pushpitha K, Joseph C, Gupta V, Rajput R, Chitranshi N et al (2019) Amyloid beta induces early changes in the ribosomal machinery, cytoskeletal organization and oxidative phosphorylation in retinal photoreceptor cells. Front Mol Neurosci 12:24. 10.3389/fnmol.2019.00024 30853886 10.3389/fnmol.2019.00024 PMC6395395

[21] Ehrlich JR, Swenor BK, Zhou Y, Langa KM (2021) The longitudinal association of vision impairment with transitions to cognitive impairment and dementia: findings from the Aging, Demographics and Memory Study. J Gerontol A Biol Sci Med Sci 76:2187–2193. 10.1093/gerona/glab157 34061956 10.1093/gerona/glab157 PMC8599065

[22] Grimaldi A, Pediconi N, Oieni F, Pizzarelli R, Rosito M, Giubettini M et al (2019) Neuroinflammatory processes, A1 astrocyte activation and protein aggregation in the retina of Alzheimer’s disease patients, possible biomarkers for early diagnosis. Front Neurosci 13:925. 10.3389/fnins.2019.00925 31551688 10.3389/fnins.2019.00925 PMC6737046

[23] Hang A, Shao A, Shea M, Roux MJ, Imai-Leonard DM, Adams DJ et al (2025) Ocular phenotyping of knockout mice identifies genes associated with late adult retinal phenotypes. Invest Ophthalmol Vis Sci 66:64. 10.1167/iovs.66.6.64 40548636 10.1167/iovs.66.6.64 PMC12186831

[24] Hart de Ruyter FJ, Morrema THJ, den Haan J, Netherlands Brain B, Twisk JWR, de Boer JF et al (2023) Phosphorylated tau in the retina correlates with tau pathology in the brain in Alzheimer’s disease and primary tauopathies. Acta Neuropathol 145:197–218. 10.1007/s00401-022-02525-1 36480077 10.1007/s00401-022-02525-1

[25] Hashimoto R, Jiang M, Shiba T, Hiruta N, Takahashi M, Higashi M et al (2017) Soluble form of LR11 is highly increased in the vitreous fluids of patients with idiopathic epiretinal membrane. Graefes Arch Clin Exp Ophthalmol 255:885–891. 10.1007/s00417-017-3585-1 28102455 10.1007/s00417-017-3585-1

[26] Hediyeh-Zadeh S, Webb AI, Davis MJ (2023) MsImpute: estimation of missing peptide intensity data in label-free quantitative mass spectrometry. Mol Cell Proteomics 22:100558. 10.1016/j.mcpro.2023.100558 37105364 10.1016/j.mcpro.2023.100558 PMC10368900

[27] Heneka MT, Golenbock DT, Latz E (2015) Innate immunity in Alzheimer’s disease. Nat Immunol 16:229–236. 10.1038/ni.3102 25689443 10.1038/ni.3102

[28] Hondius DC, van Nierop P, Li KW, Hoozemans JJ, van der Schors RC, van Haastert ES et al (2016) Profiling the human hippocampal proteome at all pathologic stages of Alzheimer’s disease. Alzheimers Dement 12:654–668. 10.1016/j.jalz.2015.11.002 26772638 10.1016/j.jalz.2015.11.002

[29] Hosono K, Ishigami C, Takahashi M, Park DH, Hirami Y, Nakanishi H et al (2012) Two novel mutations in the EYS gene are possible major causes of autosomal recessive retinitis pigmentosa in the Japanese population. PLoS ONE 7:e31036. 10.1371/journal.pone.0031036 22363543 10.1371/journal.pone.0031036 PMC3281914

[30] Is O, Wang X, Reddy JS, Min Y, Yilmaz E, Bhattarai P et al (2024) Gliovascular transcriptional perturbations in Alzheimer’s disease reveal molecular mechanisms of blood brain barrier dysfunction. Nat Commun 15:4758. 10.1038/s41467-024-48926-6 38902234 10.1038/s41467-024-48926-6 PMC11190273

[31] Jiang G, Xie G, Li X, Xiong J (2025) Cytoskeletal proteins and Alzheimer’s disease pathogenesis: focusing on the interplay with Tau pathology. Biomolecules. 10.3390/biom15060831 40563471 10.3390/biom15060831 PMC12190275

[32] Johnson ECB, Bian S, Haque RU, Carter EK, Watson CM, Gordon BA et al (2023) Cerebrospinal fluid proteomics define the natural history of autosomal dominant Alzheimer’s disease. Nat Med 29:1979–1988. 10.1038/s41591-023-02476-4 37550416 10.1038/s41591-023-02476-4 PMC10427428

[33] Koronyo Y, Biggs D, Barron E, Boyer DS, Pearlman JA, Au WJ et al (2017) Retinal amyloid pathology and proof-of-concept imaging trial in Alzheimer’s disease. JCI Insight. 10.1172/jci.insight.93621 28814675 10.1172/jci.insight.93621 PMC5621887

[34] Koronyo Y, Rentsendorj A, Mirzaei N, Regis GC, Sheyn J, Shi H et al (2023) Retinal pathological features and proteome signatures of Alzheimer’s disease. Acta Neuropathol 145:409–438. 10.1007/s00401-023-02548-2 36773106 10.1007/s00401-023-02548-2 PMC10020290

[35] La Morgia C, Ross-Cisneros FN, Koronyo Y, Hannibal J, Gallassi R, Cantalupo G et al (2016) Melanopsin retinal ganglion cell loss in Alzheimer disease. Ann Neurol 79:90–109. 10.1002/ana.24548 26505992 10.1002/ana.24548 PMC4737313

[36] Lace G, Savva GM, Forster G, de Silva R, Brayne C, Matthews FE et al (2009) Hippocampal tau pathology is related to neuroanatomical connections: an ageing population-based study. Brain 132:1324–1334. 10.1093/brain/awp059 19321462 10.1093/brain/awp059

[37] Lazar C, Gatto L, Ferro M, Bruley C, Burger T (2016) Accounting for the multiple natures of missing values in label-free quantitative proteomics data sets to compare imputation strategies. J Proteome Res 15:1116–1125. 10.1021/acs.jproteome.5b00981 26906401 10.1021/acs.jproteome.5b00981

[38] Lee S, Jiang K, McIlmoyle B, To E, Xu QA, Hirsch-Reinshagen V et al (2020) Amyloid beta immunoreactivity in the retinal ganglion cell layer of the Alzheimer’s eye. Front Neurosci 14:758. 10.3389/fnins.2020.00758 32848548 10.3389/fnins.2020.00758 PMC7412634

[39] Li J, Wang J, Ibarra IL, Cheng X, Luecken MD, Lu J et al (2026) Single-cell atlas of the transcriptome and chromatin accessibility in the human retina. Nat Genet 58:418–433. 10.1038/s41588-025-02454-1 41578023 10.1038/s41588-025-02454-1 PMC13050529

[40] Liu J, Baum L, Yu S, Lin Y, Xiong G, Chang RC et al (2021) Preservation of retinal function through synaptic stabilization in Alzheimer’s disease model mouse retina by *Lycium barbarum* extracts. Front Aging Neurosci 13:788798. 10.3389/fnagi.2021.788798 35095474 10.3389/fnagi.2021.788798 PMC8792986

[41] London A, Benhar I, Schwartz M (2013) The retina as a window to the brain-from eye research to CNS disorders. Nat Rev Neurol 9:44–53. 10.1038/nrneurol.2012.227 23165340 10.1038/nrneurol.2012.227

[42] Maes ME, Donahue RJ, Schlamp CL, Marola OJ, Libby RT, Nickells RW (2023) BAX activation in mouse retinal ganglion cells occurs in two temporally and mechanistically distinct steps. Mol Neurodegener 18:67. 10.1186/s13024-023-00659-8 37752598 10.1186/s13024-023-00659-8 PMC10521527

[43] Metri MN, You J, Park J (2025) Protocol for autofluorescence removal in microglia by photobleaching in free-floating immunofluorescent staining of mouse brain tissue. STAR Protoc 6:104199. 10.1016/j.xpro.2025.104199 41231674 10.1016/j.xpro.2025.104199 PMC12662085

[44] Mishra S, Knupp A, Szabo MP, Williams CA, Kinoshita C, Hailey DW et al (2022) The Alzheimer’s gene SORL1 is a regulator of endosomal traffic and recycling in human neurons. Cell Mol Life Sci 79:162. 10.1007/s00018-022-04182-9 35226190 10.1007/s00018-022-04182-9 PMC8885486

[45] Murphy MP, Das P, Nyborg AC, Rochette MJ, Dodson MW, Loosbrock NM et al (2003) Overexpression of nicastrin increases Abeta production. FASEB J 17:1138–1140. 10.1096/fj.02-1050fje 12692078 10.1096/fj.02-1050fje

[46] Mutlu U, Colijn JM, Ikram MA, Bonnemaijer PWM, Licher S, Wolters FJ et al (2018) Association of retinal neurodegeneration on optical coherence tomography with dementia: a population-based study. JAMA Neurol 75:1256–1263. 10.1001/jamaneurol.2018.1563 29946702 10.1001/jamaneurol.2018.1563 PMC6233847

[47] Naito Y, Tanabe Y, Lee AK, Hamel E, Takahashi H (2017) Amyloid-beta oligomers interact with neurexin and diminish neurexin-mediated excitatory presynaptic organization. Sci Rep 7:42548. 10.1038/srep42548 28211900 10.1038/srep42548 PMC5304201

[48] Negro D, Opazo P (2024) Cognitive resilience in Alzheimer’s disease: from large-scale brain networks to synapses. Brain Commun. 6:fcae050. 10.1093/braincomms/fcae050 38425748 10.1093/braincomms/fcae050 PMC10903981

[49] Nunez-Diaz C, Andersson E, Schultz N, Poceviciute D, Hansson O, Netherlands Brain B et al (2024) The fluorescent ligand bTVBT2 reveals increased p-tau uptake by retinal microglia in Alzheimer’s disease patients and App(NL-F/NL-F) mice. Alzheimers Res Ther 16:4. 10.1186/s13195-023-01375-7 38167557 10.1186/s13195-023-01375-7 PMC10763304

[50] Oh HS, Urey DY, Karlsson L, Zhu Z, Shen Y, Farinas A et al (2025) A cerebrospinal fluid synaptic protein biomarker for prediction of cognitive resilience versus decline in Alzheimer’s disease. Nat Med 31:1592–1603. 10.1038/s41591-025-03565-2 40164724 10.1038/s41591-025-03565-2 PMC12092275

[51] Perez-Riverol Y, Bandla C, Kundu DJ, Kamatchinathan S, Bai J, Hewapathirana S et al (2025) The PRIDE database at 20 years: 2025 update. Nucleic Acids Res 53:D543–D553. 10.1093/nar/gkae1011 39494541 10.1093/nar/gkae1011 PMC11701690

[52] Pescoller J, Dewenter A, Dehsarvi A, Steward A, Frontzkowski L, Zhu Z et al (2025) Cortical tau deposition promotes atrophy in connected white matter regions in Alzheimer’s disease. Brain. 10.1093/brain/awaf339 40966722 10.1093/brain/awaf339

[53] Pichet Binette A, Gaiteri C, Wennstrom M, Kumar A, Hristovska I, Spotorno N et al (2024) Proteomic changes in Alzheimer’s disease associated with progressive Abeta plaque and tau tangle pathologies. Nat Neurosci 27:1880–1891. 10.1038/s41593-024-01737-w 39187705 10.1038/s41593-024-01737-w PMC11452344

[54] Polo V, Rodrigo MJ, Garcia-Martin E, Otin S, Larrosa JM, Fuertes MI et al (2017) Visual dysfunction and its correlation with retinal changes in patients with Alzheimer’s disease. Eye (Lond) 31:1034–1041. 10.1038/eye.2017.23 28282060 10.1038/eye.2017.23 PMC5519267

[55] Program CZICS, Abdulla S, Aevermann B, Assis P, Badajoz S, Bell SM et al (2025) CZ CELLxGENE Discover: a single-cell data platform for scalable exploration, analysis and modeling of aggregated data. Nucleic Acids Res 53:D886–D900. 10.1093/nar/gkae1142 39607691 10.1093/nar/gkae1142 PMC11701654

[56] Rohden F, Ferreira PCL, Bellaver B, Ferrari-Souza JP, Aguzzoli CS, Soares C et al (2025) Glial reactivity correlates with synaptic dysfunction across aging and Alzheimer’s disease. Nat Commun 16:5653. 10.1038/s41467-025-60806-1 40593718 10.1038/s41467-025-60806-1 PMC12215965

[57] Saleem K, Xiao Z, Zhu B, Ren Y, Yan Z, Feng J (2025) Elevated SGK1 increases Tau phosphorylation and microtubule instability in Alzheimer’s patient-derived cortical neurons. Mol Psychiatry. 10.1038/s41380-025-03225-4 40921794 10.1038/s41380-025-03225-4 PMC12700806

[58] Sandebring-Matton A, Axenhus M, Bogdanovic N, Winblad B, Schedin-Weiss S, Nilsson P et al (2021) Microdissected pyramidal cell proteomics of Alzheimer brain reveals alterations in creatine kinase B-type, 14–3-3-gamma, and heat shock cognate 71. Front Aging Neurosci 13:735334. 10.3389/fnagi.2021.735334 34867272 10.3389/fnagi.2021.735334 PMC8641652

[59] Santiago J, Poceviciute D, Netherlands Brain B, Wennstrom M (2025) Perivascular phosphorylated TDP-43 inclusions are associated with Alzheimer’s disease pathology and loss of CD146 and Aquaporin-4. Brain Pathol 35:e13304. 10.1111/bpa.13304 39251230 10.1111/bpa.13304 PMC11835440

[60] Santiago J, Poceviciute D, Vogel J, Netherlands Brain B, Brinkmalm G, Wennstrom M (2025) Retinal tau phosphorylation in Alzheimer’s disease: a mass spectrometry study. Neurobiol Dis 215:107057. 10.1016/j.nbd.2025.107057 40835172 10.1016/j.nbd.2025.107057

[61] Schultz N, Byman E, Netherlands Brain B, Wennstrom M (2020) Levels of retinal amyloid-beta correlate with levels of retinal IAPP and hippocampal amyloid-beta in neuropathologically evaluated individuals. J Alzheimers Dis 73:1201–1209. 10.3233/JAD-190868 31884473 10.3233/JAD-190868 PMC7081096

[62] Schultz N, Byman E, Netherlands Brain B, Wennstrom M (2018) Levels of retinal IAPP are altered in Alzheimer’s disease patients and correlate with vascular changes and hippocampal IAPP levels. Neurobiol Aging 69:94–101. 10.1016/j.neurobiolaging.2018.05.003 29864717 10.1016/j.neurobiolaging.2018.05.003

[63] Selkoe DJ, Hardy J (2016) The amyloid hypothesis of Alzheimer’s disease at 25 years. EMBO Mol Med 8:595–608. 10.15252/emmm.201606210 27025652 10.15252/emmm.201606210 PMC4888851

[64] Shi H, Koronyo Y, Fuchs DT, Sheyn J, Jallow O, Mandalia K et al (2023) Retinal arterial Abeta(40) deposition is linked with tight junction loss and cerebral amyloid angiopathy in MCI and AD patients. Alzheimers Dement 19:5185–5197. 10.1002/alz.13086 37166032 10.1002/alz.13086 PMC10638467

[65] Shi H, Koronyo Y, Fuchs DT, Sheyn J, Wawrowsky K, Lahiri S et al (2020) Retinal capillary degeneration and blood-retinal barrier disruption in murine models of Alzheimer’s disease. Acta Neuropathol Commun 8:202. 10.1186/s40478-020-01076-4 33228786 10.1186/s40478-020-01076-4 PMC7686701

[66] Shi H, Koronyo Y, Rentsendorj A, Fuchs DT, Sheyn J, Black KL et al (2021) Retinal vasculopathy in Alzheimer’s disease. Front Neurosci 15:731614. 10.3389/fnins.2021.731614 34630020 10.3389/fnins.2021.731614 PMC8493243

[67] Shiba T, Bujo H, Takahashi M, Sato Y, Jiang M, Hori Y et al (2013) Vitreous fluid and circulating levels of soluble lr11, a novel marker for progression of diabetic retinopathy. Graefes Arch Clin Exp Ophthalmol 251:2689–2695. 10.1007/s00417-013-2373-9 23652469 10.1007/s00417-013-2373-9

[68] Sweeney MD, Sagare AP, Zlokovic BV (2018) Blood-brain barrier breakdown in Alzheimer disease and other neurodegenerative disorders. Nat Rev Neurol 14:133–150. 10.1038/nrneurol.2017.188 29377008 10.1038/nrneurol.2017.188 PMC5829048

[69] Taddei RN, K ED, (2025) Synapse vulnerability and resilience underlying Alzheimer’s disease. EBioMedicine 112:105557. 10.1016/j.ebiom.2025.105557 39891995 10.1016/j.ebiom.2025.105557 PMC11833146

[70] Takki K (1974) Gyrate atrophy of the choroid and retina associated with hyperornithinaemia. Br J Ophthalmol 58:3–23. 10.1136/bjo.58.1.3 4841281 10.1136/bjo.58.1.3 PMC1042586

[71] Thinakaran G, Koo EH (2008) Amyloid precursor protein trafficking, processing, and function. J Biol Chem 283:29615–29619. 10.1074/jbc.R800019200 18650430 10.1074/jbc.R800019200 PMC2573065

[72] Tijms BM, Vromen EM, Mjaavatten O, Holstege H, Reus LM, van der Lee S et al (2024) Cerebrospinal fluid proteomics in patients with Alzheimer’s disease reveals five molecular subtypes with distinct genetic risk profiles. Nat Aging 4:33–47. 10.1038/s43587-023-00550-7 38195725 10.1038/s43587-023-00550-7 PMC10798889

[73] Tyler SL, Maltby J, Paterson KB, Hutchinson CV (2022) Reduced vision-related quality of life in dementia: a preliminary report. J Alzheimers Dis 87:239–246. 10.3233/JAD-215435 35275536 10.3233/JAD-215435

[74] Tzioras M, McGeachan RI, Durrant CS, Spires-Jones TL (2023) Synaptic degeneration in Alzheimer disease. Nat Rev Neurol 19:19–38. 10.1038/s41582-022-00749-z 36513730 10.1038/s41582-022-00749-z

[75] Walkiewicz G, Ronisz A, Van Ginderdeuren R, Lemmens S, Bouwman FH, Hoozemans JJM et al (2024) Primary retinal tauopathy: a tauopathy with a distinct molecular pattern. Alzheimers Dement 20:330–340. 10.1002/alz.13424 37615275 10.1002/alz.13424 PMC10916964

[76] Wang YR, Zeng XQ, Wang J, Fowler CJ, Li QX, Bu XL et al (2024) Autoantibodies to BACE1 promote Abeta accumulation and neurodegeneration in Alzheimer’s disease. Acta Neuropathol 148:57. 10.1007/s00401-024-02814-x 39448400 10.1007/s00401-024-02814-x

[77] Wei Y, Du X, Guo H, Han J, Liu M (2024) Mitochondrial dysfunction and Alzheimer’s disease: pathogenesis of mitochondrial transfer. Front Aging Neurosci 16:1517965. 10.3389/fnagi.2024.1517965 39741520 10.3389/fnagi.2024.1517965 PMC11685155

[78] Xu QA, Boerkoel P, Hirsch-Reinshagen V, Mackenzie IR, Hsiung GR, Charm G et al (2022) Muller cell degeneration and microglial dysfunction in the Alzheimer’s retina. Acta Neuropathol Commun 10:145. 10.1186/s40478-022-01448-y 36199154 10.1186/s40478-022-01448-y PMC9533552

[79] Xu Y, Aung HL, Hesam-Shariati N, Keay L, Sun X, Phu J et al (2024) Contrast sensitivity, visual field, color vision, motion perception, and cognitive impairment: a systematic review. J Am Med Dir Assoc 25:105098. 10.1016/j.jamda.2024.105098 38908397 10.1016/j.jamda.2024.105098

[80] Yamazaki Y, Shinohara M, Shinohara M, Yamazaki A, Murray ME, Liesinger AM et al (2019) Selective loss of cortical endothelial tight junction proteins during Alzheimer’s disease progression. Brain 142:1077–1092. 10.1093/brain/awz011 30770921 10.1093/brain/awz011 PMC6439325

[81] Yarbro JM, Shrestha HK, Wang Z, Zhang X, Zaman M, Chu M et al (2025) Proteomic landscape of Alzheimer’s disease: emerging technologies, advances and insights (2021–2025). Mol Neurodegener 20:83. 10.1186/s13024-025-00874-5 40660303 10.1186/s13024-025-00874-5 PMC12257826

[82] Yildiz A (2025) Mechanism and regulation of kinesin motors. Nat Rev Mol Cell Biol 26:86–103. 10.1038/s41580-024-00780-6 39394463 10.1038/s41580-024-00780-6

[83] Yu Y, Chen R, Mao K, Deng M, Li Z (2024) The role of glial cells in synaptic dysfunction: insights into Alzheimer’s disease mechanisms. Aging Dis 15:459–479. 10.14336/AD.2023.0718 37548934 10.14336/AD.2023.0718 PMC10917533

[84] Yuan Y, Zhao G, Zhao Y (2024) Dysregulation of energy metabolism in Alzheimer’s disease. J Neurol 272:2. 10.1007/s00415-024-12800-8 39621206 10.1007/s00415-024-12800-8 PMC11611936

