# Supplementary material for: Retinal proteome changes mirror brain pathology and reveal synaptic and cytoskeletal dysfunction in Alzheimer’s disease
Source: Acta Neuropathol. 2026 Jul 29;152(1):12. doi: 10.1007/s00401-026-03054-x (PMC13421282; doi:10.1007/s00401-026-03054-x)
Supplement: Supplementary file 1 — Supplementary file1 (DOCX 3973 KB) [file 401_2026_3054_MOESM1_ESM.docx]

**Supplementary Figure 1.** Cell-type enrichment of the most significantly up- and down-regulated retinal proteins in Alzheimer’s disease


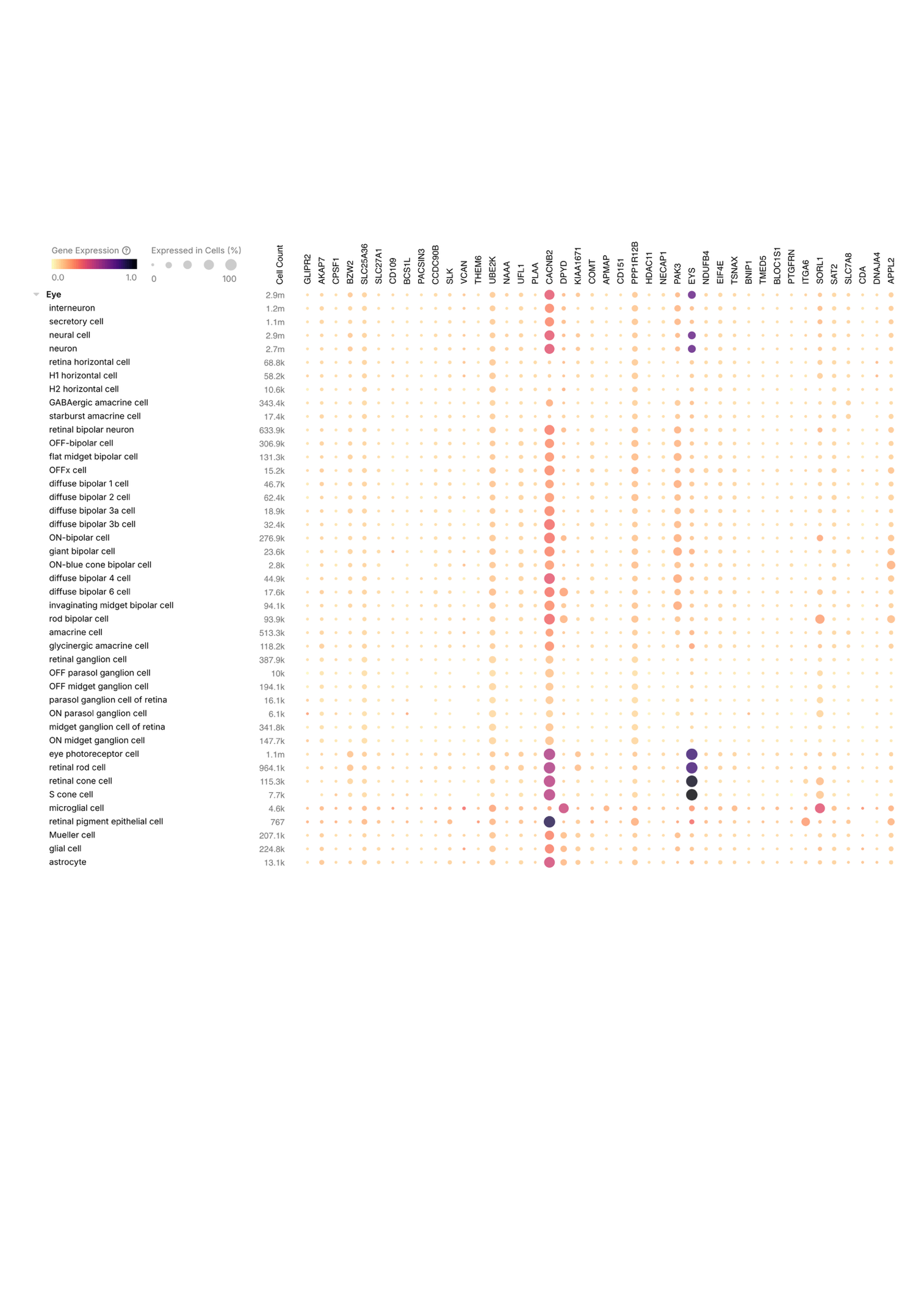


Dot plot showing expression patterns of the most significantly up- and down-regulated differentially abundant proteins (DAPs) across retinal cell populations. Dot size represents the percentage of cells expressing each plotted gene within a given cell type, and color intensity indicates normalized expression relative to all genes shown in the plot (scale bar, top right). Analysis was performed using single-cell transcriptomic reference data from the Human Cell Atlas Retina v1.0.

**Supplementary Figure 2.** STRING interaction network and functional clustering of differentially abundant proteins in the AD retina.

a.


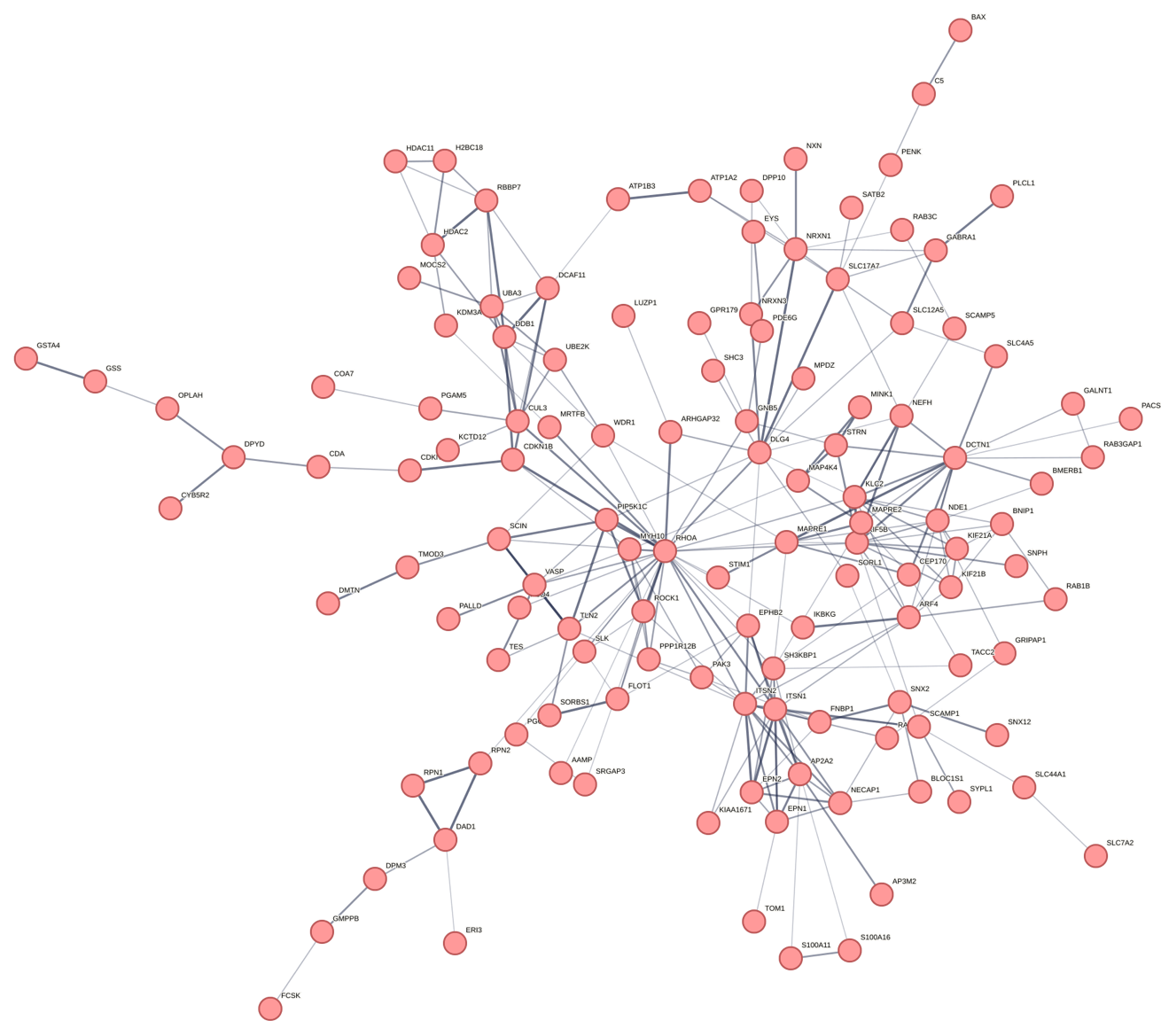


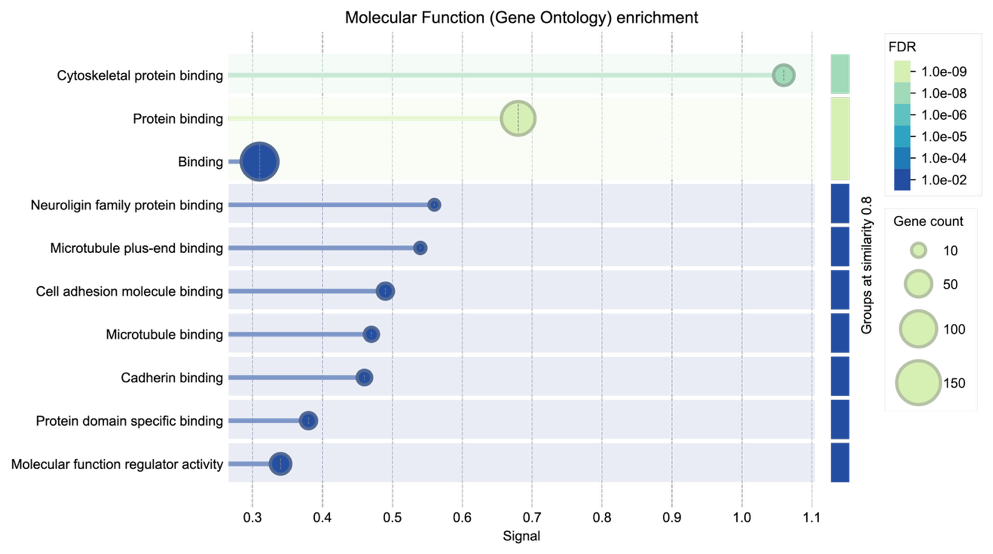


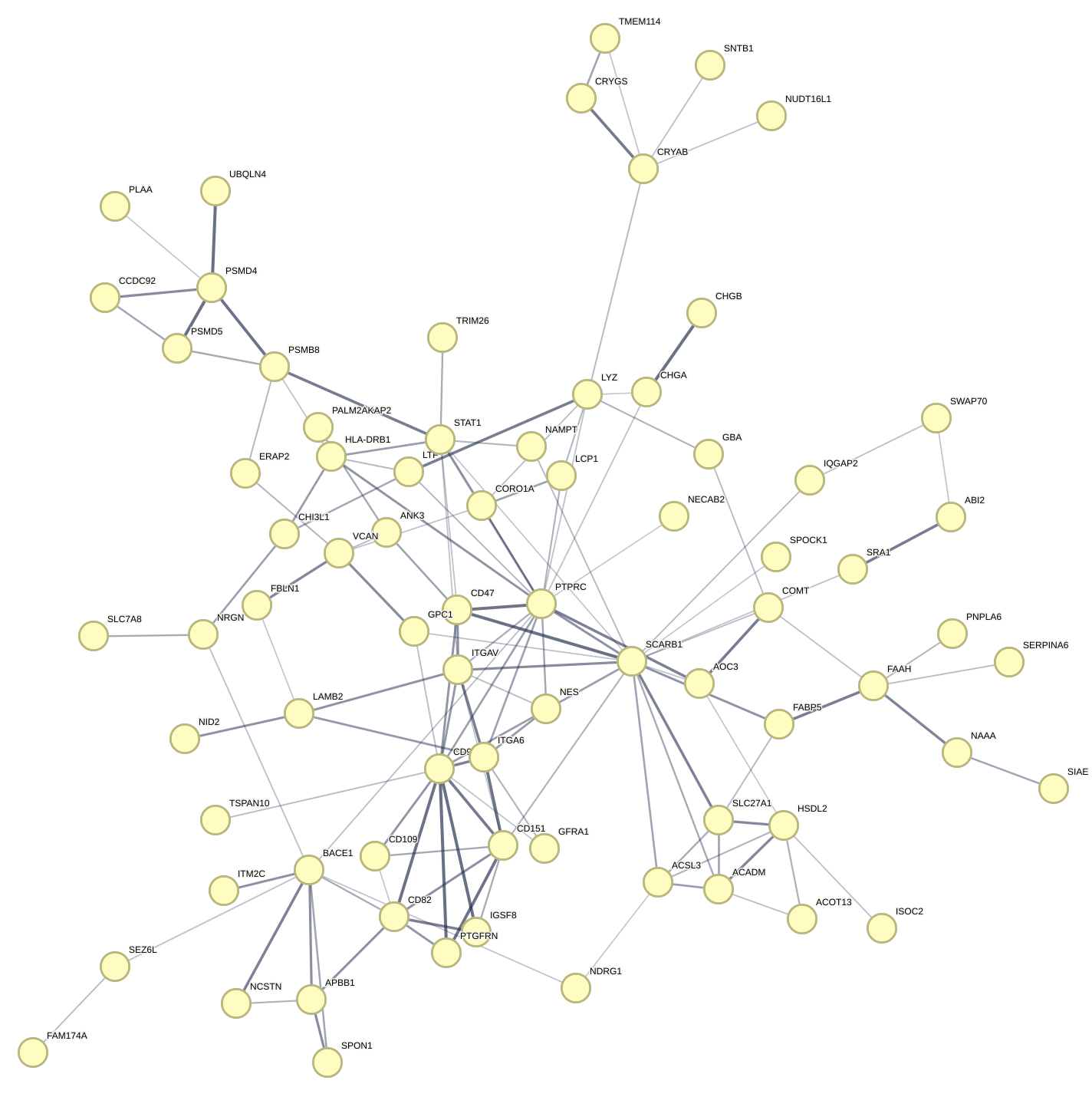
b.


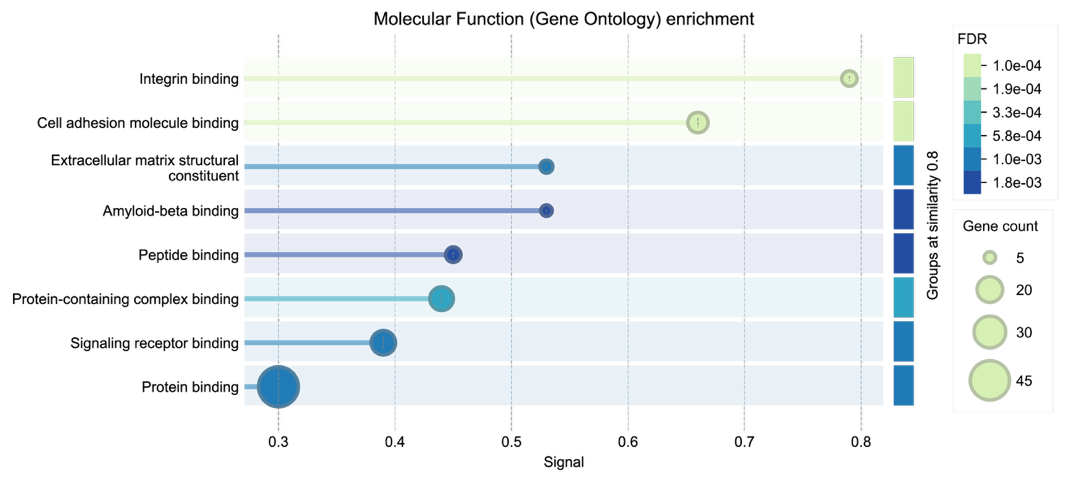


c.


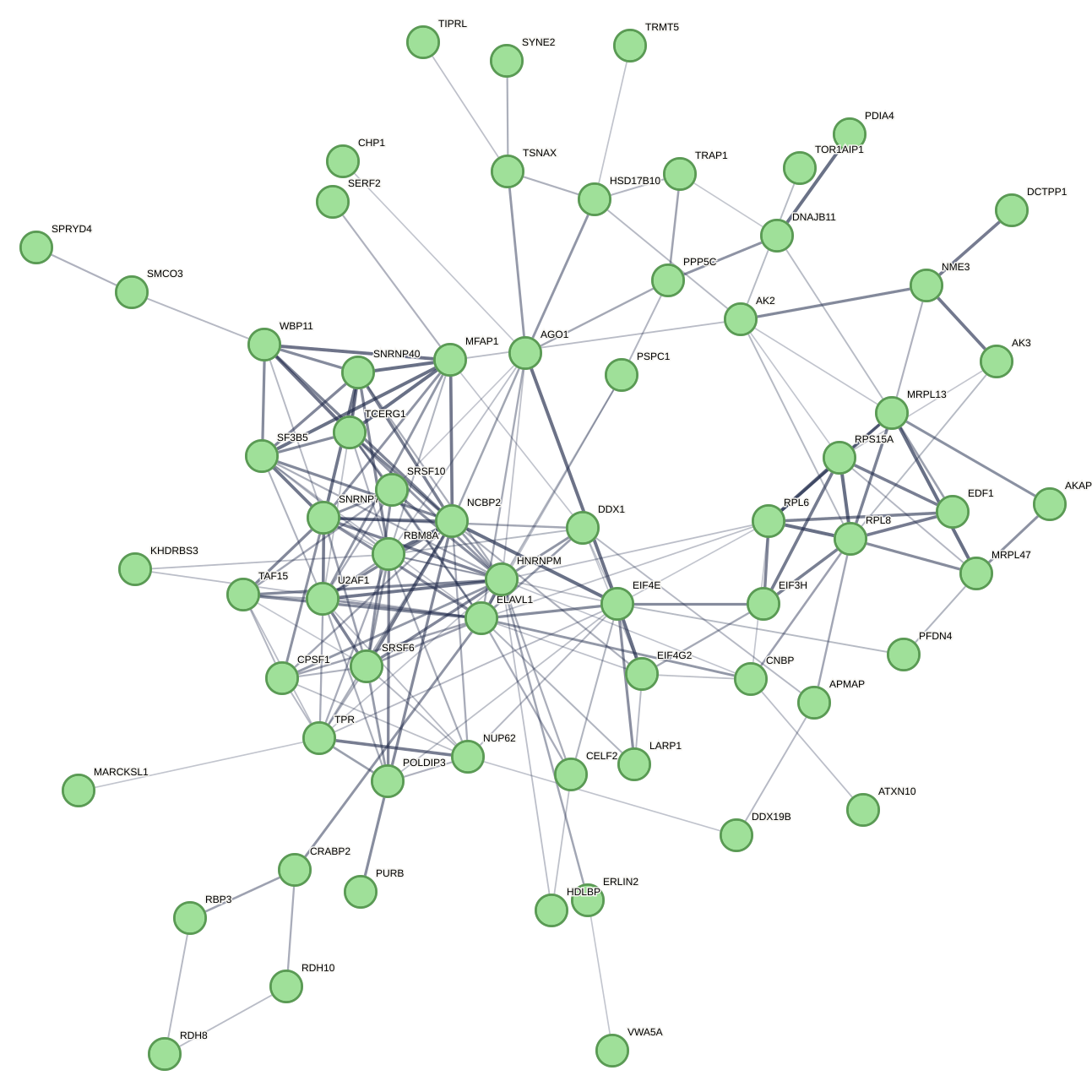


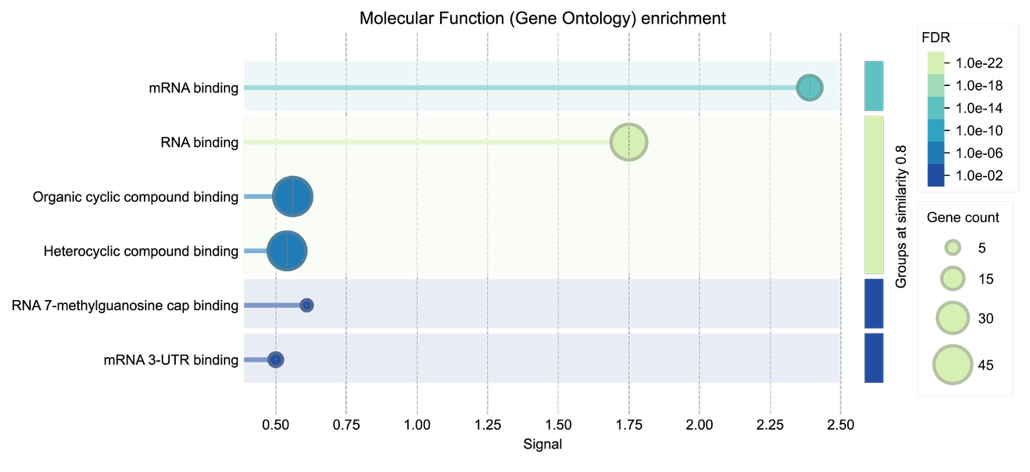


d.
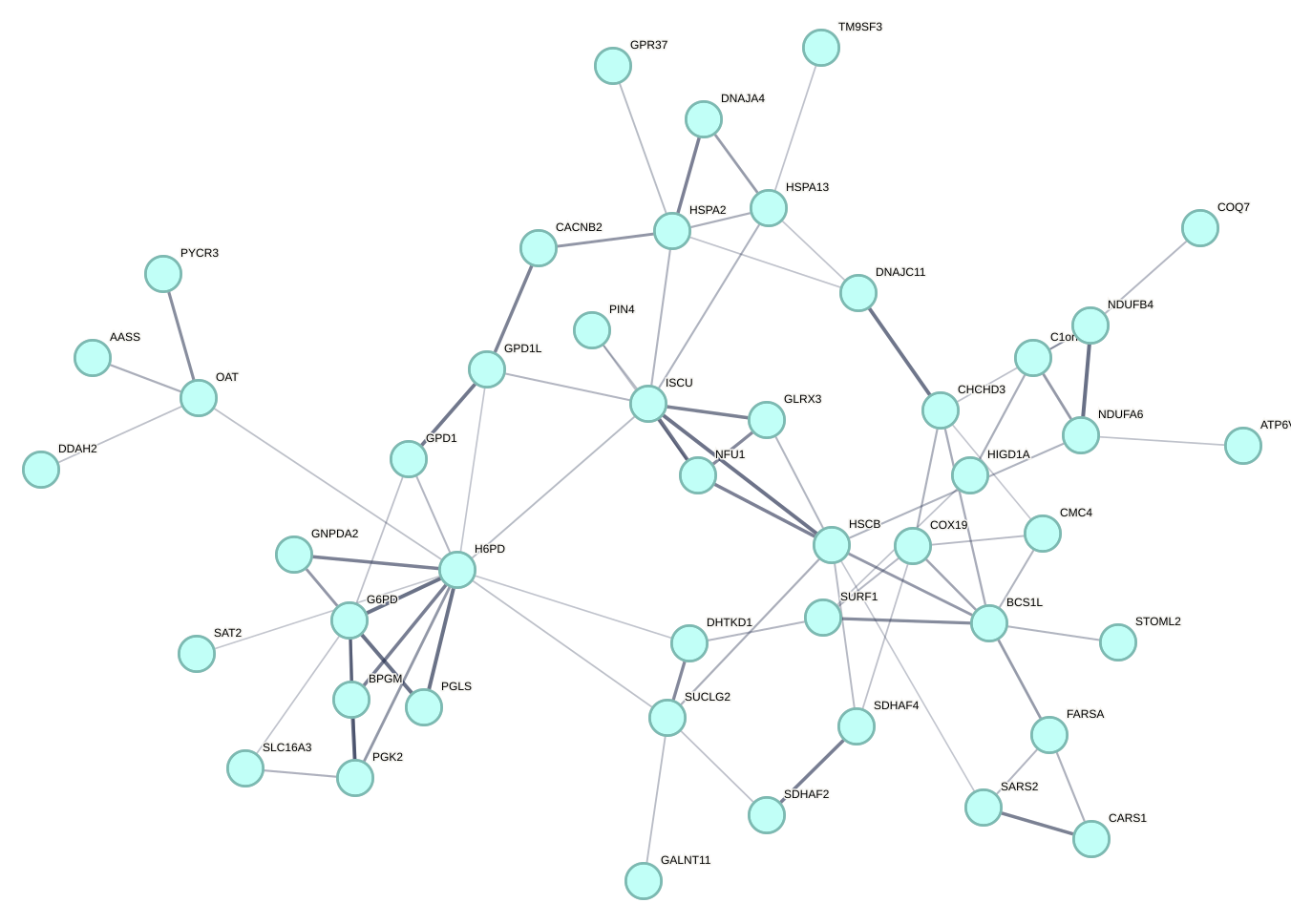


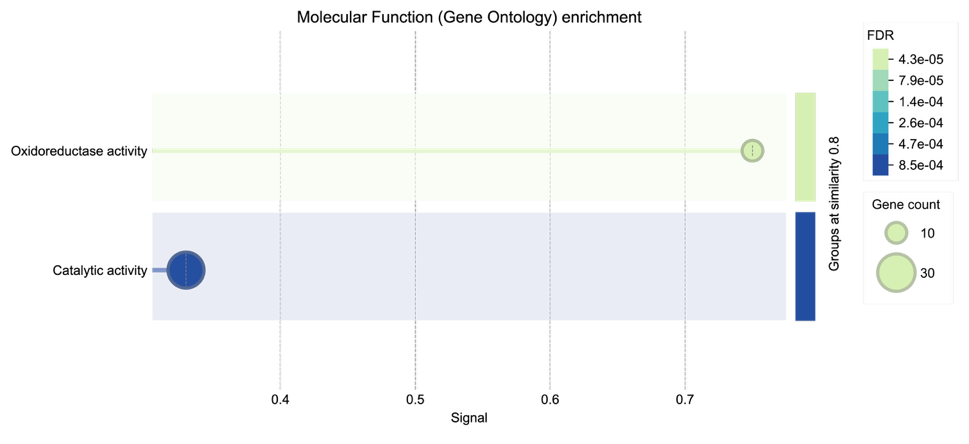


Protein–protein interaction network of all differentially abundant proteins (DAPs) identified in the AD retina, constructed using the STRING database (v12.0) with default settings (minimum interaction score 0.4; all interaction sources active). Nodes represent individual proteins and edges represent predicted functional interactions. The network was partitioned into five clusters using k-means clustering as implemented in the STRING interface. Panels (a–d) show each cluster individually with the top enriched Gene Ontology Molecular Function terms listed below each panel. (a) Cluster 1: Cytoskeletal protein binding; (b) Cluster 2: Integrin and Amyloid-beta binding; (c) Cluster 3: mRNA and RNA binding ; (d) Cluster 4: Oxidoreductase activity. Cluster 5 contains two proteins CHMP4A and VPS4A; no statistically significant molecular function enrichment was identified for this cluster. Node color corresponds to cluster identity. Edge thickness indicates interaction confidence score

**Supplementary Figure 3:** Buffer-specific distribution of differentially abundant proteins in the AD hippocampus.


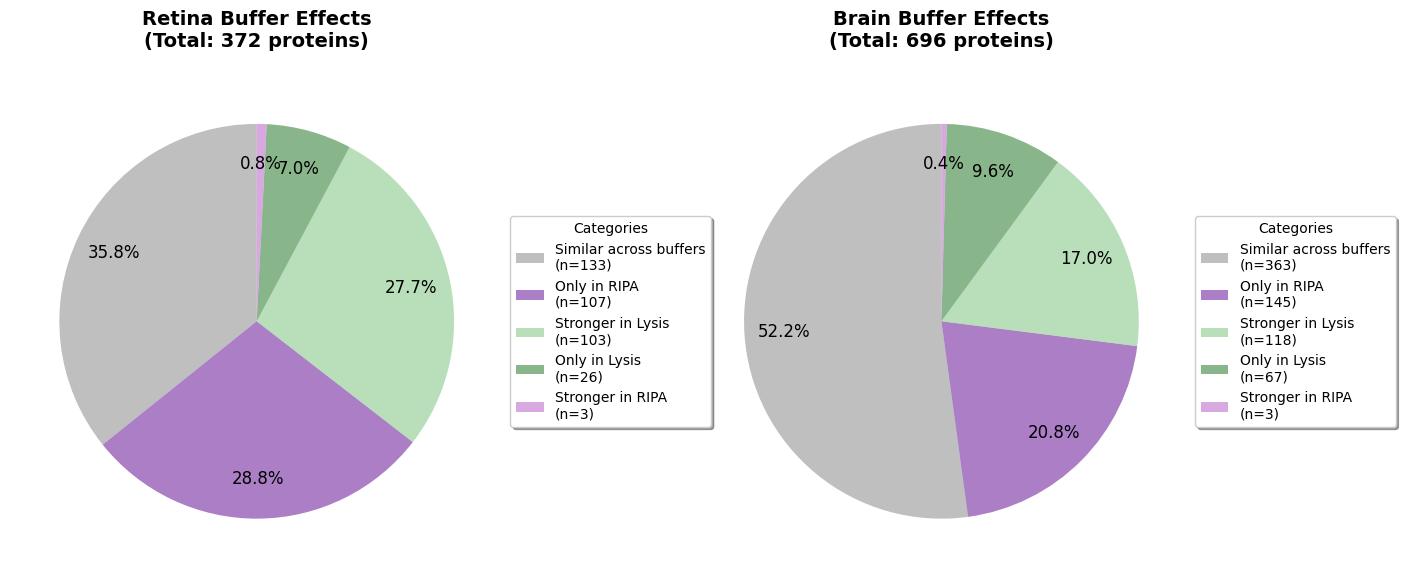


Pie chart showing the proportion of differentially abundant proteins (DAPs; total n = 696) classified by their extraction buffer profile in the hippocampus. "Similar across buffers" indicates proteins with a consistent main effect of diagnosis regardless of extraction condition. "Only in RIPA" and "Only in Lysis" denote proteins reaching statistical significance exclusively in the respective fraction. "Stronger in RIPA" and "Stronger in Lysis" denote proteins with a significant Diagnosis × Buffer interaction, indicating a significantly larger effect size in that fraction. The majority of DAPs showed consistent changes across both buffers (52.2%), while a substantial proportion were detected exclusively or more strongly in a specific fraction, suggesting differences in protein solubility or subcellular compartmentalization.

**Supplementary Figure 4. Immunofluorescence analysis of APMAP and PACSIN3 in control and Alzheimer's disease retinas.**


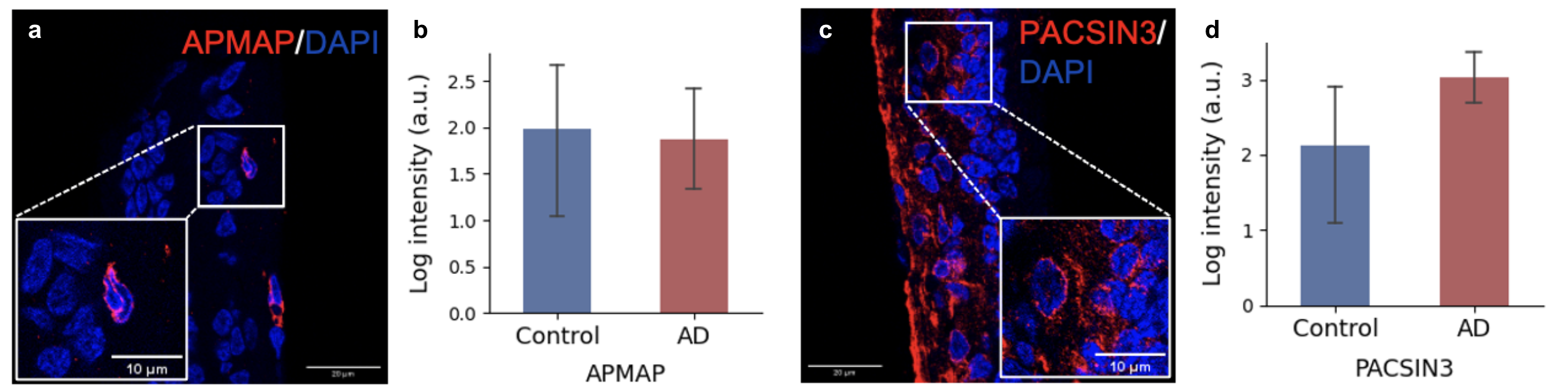


(a) Representative immunofluorescence image showing APMAP (red) and DAPI nuclear stain (blue) in retinal tissue. Insets show higher magnification views of APMAP localization within retinal cells. Scale bars: 20 μm (main image) and 10 μm (insets). (b) Quantification of APMAP immunofluorescence signal intensity (log-transformed) comparing control (ND, n=6, blue) and Alzheimer's disease (AD, n=6, red) retinal samples. No statistically significant difference was observed between groups (mean ± SD shown). (c) Representative immunofluorescence image of PACSIN3 (red) and DAPI (blue) in retinal tissue. Insets show higher magnification views of PACSIN3 distribution in retinal cells. Scale bars: 20 μm (main image) and 10 μm (insets). (d) Quantification of PACSIN3 immunofluorescence signal intensity (log-transformed) in control (ND, n=6) and AD (n=6) retinal samples. No statistically significant difference was detected between groups (mean ± SD shown).
